# Coating Dormant Collagenase‐Producing Bacteria with Metal‐Anesthetic Networks for Precision Tumor Therapy

**DOI:** 10.1002/advs.202407402

**Published:** 2024-09-18

**Authors:** Qiuju Han, Fengmin Yang, Mian Chen, Mengmeng Zhang, Lu Wang, Hongxia Wang, Jinyao Liu, Zhenping Cao

**Affiliations:** ^1^ Shanghai Key Laboratory for Nucleic Acid Chemistry and Nanomedicine Institute of Molecular Medicine State Key Laboratory of Systems Medicine for Cancer Shanghai Cancer Institute Renji Hospital School of Medicine Shanghai Jiao Tong University Shanghai 200127 China; ^2^ School of Chemistry and Chemical Engineering Shanghai Jiao Tong University Shanghai 200240 China; ^3^ Department of Medical Oncology Fudan University Shanghai Cancer Center Department of Oncology Shanghai Medical College Fudan University Shanghai 200032 China

**Keywords:** bacteria, collagenase, metal‐phenolic networks, surface modification, tumor matrix

## Abstract

Tumor malignancy highly depends on the stiffness of tumor matrix, which mainly consists of collagen. Despite the destruction of tumor matrix is conducive to tumor therapy, it causes the risk of tumor metastasis. Here, metal‐anesthetic network‐coated dormant collagenase‐producing *Clostridium* is constructed to simultaneously destruct tumor matrix and inhibit tumor metastasis. By metal‐phenolic complexation and *π*–*π* stacking interactions, a Fe^3+^‐propofol network is formed on bacterial surface. Coated dormant *Clostridium* can selectively germinate and rapidly proliferate in tumor sites due to the ability of carried Fe^3+^ ions to promote bacterial multiplication. Intratumoral colonization of *Clostridium* produces sufficient collagenases to degrade tumor collagen mesh and the loaded propofol restrains tumor metastasis by inhibiting tumor cell migration and invasion. Meanwhile, the delivered Fe^3+^ ions are reduced to the Fe^2+^ form by intracellular glutathione, thereby inducing potent Fenton reaction to trigger lipid peroxidation and ultimate ferroptosis of tumor cells. In addition to a satisfactory safety, a single intratumoral injection of coated dormant *Clostridium* not only effectively retards the growth of established large primary tumors, but also significantly suppresses distal lung metastasis in two different orthotopic tumor models. This work proposes a strategy to develop advanced therapeutics for malignant tumor treatment and metastasis prevention.

## Graphical abstract

Coating dormant collagenase‐producing bacteria with metal‐anesthetic networks is reported to develop living anticancer therapeutics, which can germinate and proliferate in tumor sites, produce sufficient collagenases to degrade tumor matrix, restrain tumor cell migration and invasion, and induce Fenton reaction to trigger tumor cell ferroptosis. This work offers an insight to prepare multimodal bacteria‐based therapeutics for tumor treatment and metastasis prevention.

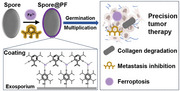

## 1 Introduction

Malignant tumors are typically incurable and life‐threatening as they grow rapidly and present a high risk of recurrence after surgery.^[^ ^1^, ^2^ ^]^ The degree of malignancy is directly associated with tumor firmness, in which augmented stiffness can promote tumor progression.^[^ ^3^, ^4^ ^]^ It has been reported that collagen, a major component of extracellular matrix, largely determines the stiffness of tumors and contributes to tumor malignancy.^[^ ^5^, ^6^, ^7^ ^]^ Cross‐linking of type I collagen alpha (COL1A) with other proteins can disrupt epithelial cadherin complex, leading to decreased intercellular contact and provoked tumor cell proliferation.^[^ ^8^ ^]^ Recently, the delivery of collagenases to tumor sites has been applied to degrade intratumoral collagen to suppress tumor firmness‐associated progression, which is conducive to tumor therapy.^[^ ^9^ ^]^ For instance, type I collagenase is encapsulated into liposomes and released in the tumor microenvironment for treating pancreatic ductal adenocarcinoma.^[^ ^10^ ^]^ Despite the achieved advancements, the in vivo application of collagenases always suffers from low drug‐loading, short half‐life, and limited intratumoral penetration.^[^ ^11^, ^12^, ^13^ ^]^ Particularly, the off‐target effect can results in severe toxicity as non‐specific degradation of systemic collagen may cause potential hemorrhaging in health organs, largely impeding the utilization of collagenases to facilitate tumor therapy.^[^ ^14^ ^]^

Owing to the innate preference for hypoxic environments, bacteria possess favorable tumor targeting, penetration, and colonization capabilities, enabling them to be a promising living therapeutic candidate for tumor treatment.^[^ ^15^, ^16^ ^]^ Bacteria can continuously express specific enzymes at tumor sites by virtue of their natural vitality, which may avoid the rapid clearance after administration and circumvent the need for repeated administration.^[^ ^17^ ^]^ Moreover, persistent bacterial growth provide the convenience for delivering sufficient cargos at the sites of interest.^[^ ^18^ ^]^ Therefore, to hinder the advancement of stubborn tumors, leveraging bacteria to produce collagenases intratumorally holds immense potential to assist tumor treatment.^[^ ^19^, ^20^ ^]^ However, the abrupt disintegration of tumor matrix adversely promotes tumor cell migration through basement membranes and ultimately causes tumor metastasis.^[^ ^21^, ^22^, ^23^ ^]^ For example, a meta‐analysis of 1569 patients with endometrial cancer displays that the high expression of matrix metalloproteinase‐2, an endogenous collagenases secreted by fibroblasts, is closely associated with tumor invasion and metastasis.^[^ ^24^ ^]^ As such, methods capable of functionalizing collagenase‐producing bacteria with an extra ability to inhibit tumor cell invasion are highly desirable for constructing bacteria‐based therapeutics that can simultaneously destruct tumor matrix to assist treatment and suppress the associated metastasis.

In the past few years, cell surface engineering, especially through chemical modification, has emerged as a facile approach to functionalize cells with augmented bioactivities and/or diverse exogenous properties.^[^ ^25^, ^26^ ^]^ Surface‐engineered cells, such as cargo‐carried red blood cells, platelets, and neutrophils as well as receptor/ligand‐anchored macrophages, dendritic cells, and T lymphocytes, have been widely explored as enhanced cell‐based therapeutics for treating different diseases.^[^ ^27^, ^28^, ^29^ ^]^ More recently, we have reported a strategy of surface coating of bacteria as a feasible yet convenient way to reinforce bacterial bioactivities and introduce coating‐derived functional motifs.^[^ ^30^, ^31^ ^]^ By tailoring the structure of the coatings, surface‐coated bacteria can be exploited as attractive living therapeutics, showing the abilities to adapt to in vivo environments and actively interact with a variety of biointerfaces.^[^ ^32^ ^]^ For instance, beneficial bacteria coated with self‐assembled lipid membranes can resist gastrointestinal insults following oral ingestion,^[^ ^33^ ^]^ while oncolytic bacteria decorated with an immunoactive polydopamine coating can provoke tumor antigen‐specific immune responses after systemic injection.^[^ ^34^ ^]^ Thus, we speculate that modifying collagenase‐expressing bacteria with a dual‐drug loaded coating may offer an opportunity to synchronously facilitate tumor therapy and prevent the occurrence of metastasis.

Here, we describe coating dormant collagenase‐producing bacteria with metal‐anesthetic networks for precision tumor therapy. By metal‐phenolic complexation and *π*–*π* stacking interactions, a Fe^3+^‐propofol network can be formed on individual dormant collagenase‐producing *Clostridium*. The resulting bacteria, in which both components of the coating are therapeutic agents, are employed to simultaneously degrades tumor matrix to assist tumor treatment and inhibits tumor metastasis. We show that coated dormant *Clostridium* selectively germinate in tumor sites due to the presence of hypoxic immunosuppressive microenvironment and efficiently proliferate given the ability of the carried Fe^3+^ ions to promote bacterial multiplication. Persistent colonization of *Clostridium* expresses sufficient collagenases to destruct intratumoral collagen mesh, while the loaded propofol prevents tumor metastasis by attenuating tumor cell migration and invasion. As the reduction of Fe^3+^ ions to the Fe^2+^ state by high‐level intracellular glutathione (GSH), the delivered Fe^3+^ ions are able to initiate potent Fenton reaction to cause lipid peroxidation and ultimate ferroptosis of tumor cells. We demonstrate that in addition to a high tolerance, a single intratumoral dose of coated dormant *Clostridium* not only significantly delays the growth of established primary tumors with a large size of ≈300 mm^3^, but also potently inhibits distal lung metastasis in two different orthotopic models of 4T1 tumor. In light of the flexibility to tune the structure and functionality of the coatings, we anticipate that this work offers an insight to prepare multimodal bacteria‐based therapeutics that can precisely treat malignant tumors and prevent tumor metastasis.

## 2 Results and Discussion

### 2.1 Design, Preparation, and Characterization of Coated Dormant *Clostridium*

**Figure 1:**
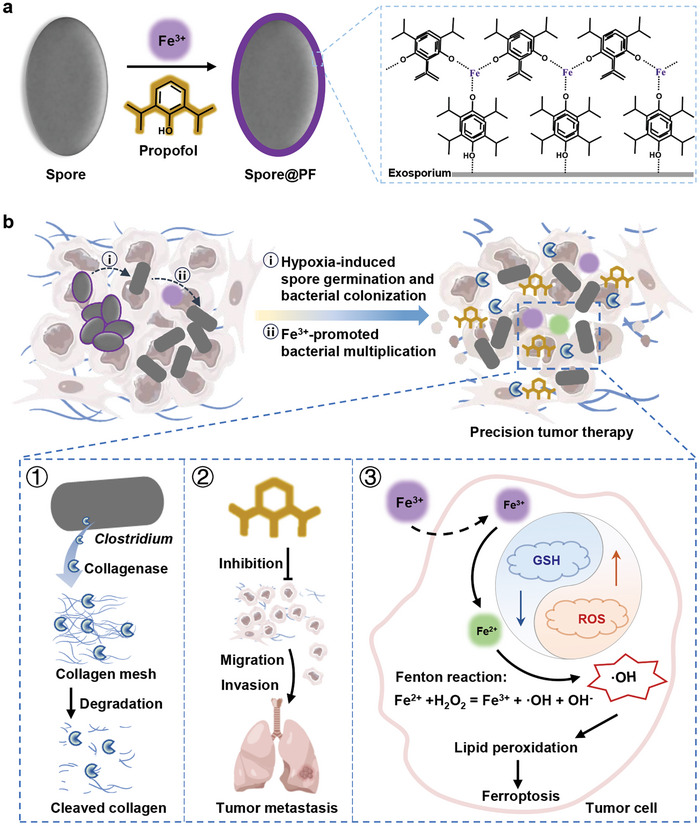
Schematic illustration. a) Preparation of Spore@PF by combining metal‐phenolic complexation and *π*–*π* stacking interactions. b) Spore@PF‐mediated degradation of intratumoral collagen mesh, suppression of tumor metastasis, and induction of tumor cell ferroptosis.

For proof of principle, a type I collagenase‐producing *Clostridium* strain was screened by using skim milk plates.^[^ ^35^ ^]^ Given the vulnerability of anaerobic bacteria as well as potential safety issues caused by the invasion and translocation of living bacteria to healthy tissues, we chose *Clostridium* in the spore form (termed Spore) for this study. Propofol, an anesthetic for patients undergoing tumor removal surgery, was selected as one of the coating components because of its ability to inhibit tumor cell migration and invasion via blocking the transmission of calcium ion channel signals.^[^ ^36^, ^37^ ^]^ Fe^3+^ ion was utilized as another component for coating formation considering its activity to induce tumor cell ferroptosis.^[^ ^38^ ^]^ Intriguingly, due to the presence of a phenolic hydroxyl group and a phenyl structure, propofol could form a network with Fe^3+^ ion on bacterial surface through metal‐phenolic complexation and π‐π stacking interactions (**Figure** **1**). The resulting Fe^3+^‐propofol network‐coated collagenase‐producing *Clostridium* spores were defined as Spore@PF.

**Figure 2:**
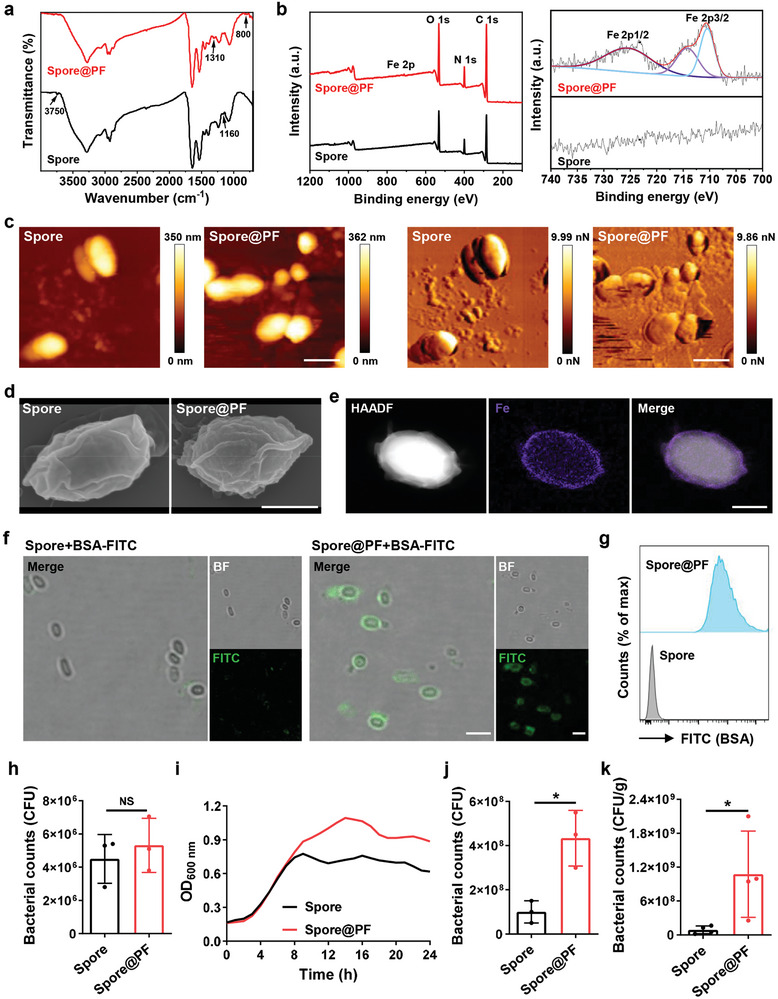
Characterization of Spore@PF. a) FT‐IR spectra of Spore and Spore@PF. b) XPS full‐survey spectra (left) and XPS deconvoluted scans (right) of Spore and Spore@PF. c) High‐resolution topography (left) and force volume (right) AFM images of Spore and Spore@PF. Scale bars, 1 µm. d) SEM images of Spore and Spore@PF. Scale bar, 500 nm. e) High‐angle annular dark‐field (HAADF)‐TEM image and corresponding EDS elemental mapping of Spore@PF. Purple indicates the distribution of Fe. Scale bar, 500 nm. f) Confocal images of Spore@PF, in which the coating was labeled with BSA‐FITC (green). The mixture of Spore and BSA‐FITC was used as a control. BF represents bright field. Scale bars, 5 µm. g) Flow cytometric analyses of Spore and Spore@PF. The coating was labeled with BSA‐FITC. h) Numbers of Spore and Spore@PF cultured on DCA plates at 37 °C under an anaerobic condition (*n* = 3). NS represents no significance. i) Growth curves of bacteria germinated from Spore or Spore@PF in Brain Heart Infusion (BHI) medium at 37 °C under an anaerobic condition. Curves were fitted using Prism (GraphPad). j) Bacterial counts of Spore and Spore@PF after 24 h culture in BHI medium under an anaerobic condition (*n* = 3). k) Numbers of germinated spores in the tumor site 24 h post intratumoral injection with Spore or Spore@PF. A number of 1 × 10^8^ CFU of spores were injected intratumorally once the size of the tumor reaching 100 mm^3^ (*n* = 4). Data are mean ± SD. Statistical analysis was assessed using two‐tailed Student's *t*‐test, giving *p* values (*p < 0.05).

Note that Spore@PF were easily prepared by co‐incubating Spore with the mixture of FeCl_3_ and propofol for 1 min at room temperature (RT). Spore@PF were first characterized by Fourier‐transform infrared (FT‐IR) spectroscopy and X‐ray photoelectron spectroscopy (XPS). As shown in **Figure** **2**a, the absorption peaks of FT‐IR at 3750 cm^−1^ (O─H stretching) and 1160 cm^−1^ (phenol C─O stretching) were absent in the spectrum of Spore@PF, while the absorption peaks assigned to CH_2_ units in Fe^3+^‐propofol network and aromatic C─H in propofol were separately observed at 1310 and ≈800 cm^−1^, indicating the formation of the coating. XPS analysis showed that stronger O1s and C1s signals were found on the spores after coating (Figure 2b). More importantly, iron ion signals (Fe 2p_1/2_ at 724 eV and Fe 2p_3/2_ at 711 eV) were observed in the spectrum of Spore@PF rather than Spore (Figure 2b). These variations suggested that both propofol and iron ions were present on Spore@PF. To directly identify the presence of the coating on spore surface, atomic force microscopy (AFM) was used to detect the biophysical surface of Spore@PF. As shown in Figure 2c, Spore@PF displayed an increment of 20–50 nm in height in comparison to that of Spore, which was attributed to the formed coating. The corresponding force‐volume images showed a higher force value of Spore than that of Spore@PF, indicating the reduced surface stiffness of Spore@PF. Compared to the uniform surface structure of Spore, there was a heterogeneous outer layer on Spore@PF under transmission electron microscopy (TEM) observation (Figure S1, Supporting Information). Similarly, as depicted in scanning electron microscope (SEM) images, Spore@PF showed a rougher surface compared to that of Spore (Figure 2d). Furthermore, the formation of an entire Fe^3+^‐propofol coating on Spore@PF was confirmed by energy dispersive X‐ray spectroscopy (EDS) mapping analysis (Figure 2e; Figure S2, Supporting Information), which displayed a uniform iron‐based shell on the surface. This core‐shell structure was also confirmed by confocal imaging, showing a fluorescent circle around the spores after staining the coating with fluorescein isothiocyanate‐labeled bovine serum albumin (BSA‐FITC) (Figure 2f). Flow cytometric analysis suggested that the spores could be quantitatively coated by simple co‐incubation (Figure 2g). In addition, we calculated the contents of propofol and Fe^3+^ ions in the coating by both ultraviolet‐visible (UV–vis) spectroscopy (Figure S3, Supporting Information) and inductively coupled plasma (ICP) test. The results revealed that 1 × 10^9^ colony forming unit (CFU) of the spores were loaded with ≈800 µg propofol and ≈85 µg iron ions, respectively.

### 2.2 Promoted Proliferation and Selective Intratumoral Germination

To assess whether the vitality of the spores was influenced after coating, equivalent Spore and Spore@PF were spread onto deoxycholate citrate agar (DCA) plates, which were subsequently cultured under an anaerobic condition at 37 °C for 24 h. Bacterial plate counting results verified a comparable number of vital bacteria between the Spore and Spore@PF groups, implying negligible impact of the coating on spore germination (Figure 2h). Meanwhile, the growth curves were monitored by recording the optical density at 600 nm (OD_600 nm_) of the culture medium. Remarkably, compared to the Spore group, we found a significant increase in bacterial growth in the Spore@PF group (Figure 2i). As quantified by plate counting, Spore@PF exhibited a 3‐fold increment in bacterial proliferation in contrast to uncoated spores (Figure 2j). The greatly promoted proliferation of the spores could be explained by the carrying of Fe^3+^ ions as previous findings have reported that iron ions are essential for bacterial multiplication.^[^ ^39^, ^40^ ^]^ To verify this, different concentrations of iron ions were added directly into the culture medium. As plotted in Figure S4 (Supporting Information), the addition of iron ions could expedite the growth of uncoated spores. Specifically, with the help of a concentration of 1 mm Fe^3+^ ions, the number of bacteria after 24 h incubation was increased by ≈20‐fold compared to the untreated group, which in turn demonstrated the beneficial effect of carried iron ions on bacterial proliferation. To further evaluate the degradability of Fe^3+^‐propofol network on spore surface, we incorporated doxycycline as a fluorescence probe in the network and monitored its release during bacterial proliferation. As shown in Figure S5 (Supporting Information), the release was negligible in phosphate buffer saline (PBS), yet ≈75% of doxycycline was released after 1 h incubation in simulated tumor microenvironment. These data suggested that the network could degrade rapidly in the tumor microenvironment due to bacterial proliferation and GSH‐responsive disassembly of Fe^3+^‐propofol network. To examine whether this promotion effect occurred in vivo, 4T1 tumor‐bearing mice were intratumorally injected with 1 × 10^8^ CFU of Spore or Spore@PF once the size of tumors reaching 100 mm^3^ and the tumor tissues were harvested to quantify the number of viable bacteria 24 h after injection. As evidenced in Figure 2k, the Spore@PF group showed a significantly increased bacterial quantity in the tumor tissue, which was ≈25‐times higher than that of the Spore group. In addition, undetectable cytotoxicity against epithelial cells was observed even with the ratio of spores to cells increasing up to 100, suggesting a favorable biosafety of Spore@PF (Figure S6, Supporting Information). Notably, negligible viable bacteria were distributed in the major organs including heart, liver, spleen, lung, and kidney of the mice following intratumoral injection, while ≈99% detected bacteria were concentrated in the tumor site (Figure S7, Supporting Information). These results illustrated that Spore@PF not only germinated specifically in the hypoxic tumor tissue, but also proliferated rapidly upon germination.

### 2.3 Ability of Spore@PF to Degrade Collagen

**Figure 3:**
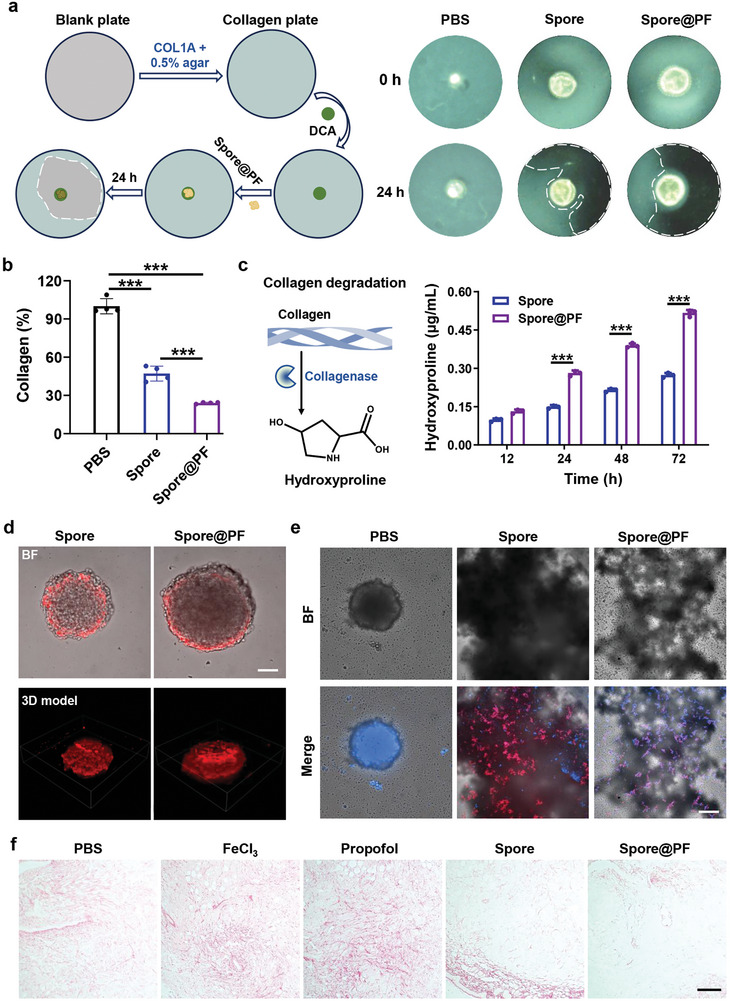
Collagen degradation mediated by Spore@PF. a) Schematic illustration (left) and digital images of typical COL1A‐containing plates after incubation with the spores for 24 h (right). White dotted lines delineate the degraded area. b) Relative collagen expression of NIH‐3T3 cells after co‐incubation with Spore or Spore@PF for 24 h by Sirius scarlet staining. Cells treated with PBS were employed as the control for normalization (*n* = 4). c) Relative hydroxyproline concentration after co‐incubation 2 × 10^6^ NIH‐3T3 cells with 2 × 10^7^ CFU of Spore or Spore@PF (MOI: 10) for the indicated time points (*n* = 4). MOI (multiplicity of infection) indicates the ratio of spores to cells. Confocal images of tumor spheres after co‐culture with Cy5.5‐labeled Spore or Spore@PF for d) 4 and e) 24 h, respectively. Red represents Cy5.5 and blue indicates cell nuclei stained with 4′,6‐diamidino‐2‐phenylindole (DAPI). Scale bars, 100 µm. f) Sirius scarlet staining of tumor tissues sampled from mice 48 h after various treatments. Scale bar, 200 µm. All data are mean ± SD. Statistical analysis was assessed using one‐way ANOVA plus Turkey's post‐test for Sirius scarlet staining and multiple Student's *t*‐test for hydroxyproline concentration, giving *p* values (***p < 0.001).

To test the collagen degradation ability of bacteria germinated from Spore@PF, COL1A was collected from rat tails. Coomassie blue staining indicated that the isolated COL1A had a comparable purity to the commercial ones (Figure S8, Supporting Information). Spore or Spore@PF pre‐cultured overnight on DCA plates were transferred to culture plates containing 10 mg mL^−1^ collagen and 0.5% agarose. Visually, the collagen plates turned to transparent after incubation with Spore or Spore@PF for 24 h under an anaerobic condition (**Figure** **3**a), suggesting the degradation of collagen by germinated spores. In comparison to Spore, more obvious hyalinization was observed in the Spore@PF group, which might be ascribed to superior bacterial proliferation enabled by the coating. Next, NIH‐3T3 fibrocytes, the major collagen‐producing cells,^[^ ^41^ ^]^ were used to assess the collagen degradation ability of the spores. After 24 h co‐incubation, Sirius scarlet staining showed enhanced collagen degradation ability of the coated spores, as reflected by the observation of ≈50% and ≈80% collagen degradation by 1 × 10^7^ CFU mL^−1^ of Spores and Spore@PF, respectively (Figure 3b). We also quantified the efficiency of collagen degradation by using a more sensitive hydroxyproline test kit, because collagen can be degraded to hydroxyproline by collagenases.^[^ ^42^ ^]^ As expected, in contrast to Spore, a higher increase in the level of hydroxyproline over time was observed in the Spore@PF group (Figure 3c). After 72 h incubation, the generation of hydroxyproline was increased by 2‐times by Spore@PF. Furthermore, 4T1 tumor spheres were established to investigate the capacity of Spore@PF to degrade tumor‐associated collagen. After 4 h co‐cultivation with Cy5.5‐labeled Spore or Spore@PF, confocal images displayed substantial invasion of the spores into the core of the tumor spheres (Figure 3d). Owing to spore germination and the rapid growth of germinated spores, the tight tumor spheres were digested into a loose and irregular‐shaped morphology. Particularly, compared to Spore, co‐culture with Spore@PF resulted in a more incompact structure of the tumor spheres, reflecting the ability to destroy tumor matrix (Figure 3e). In addition, an orthotopic 4T1 tumor model with an average tumor size of 100 mm^3^ was developed to evaluate the performance of Spore@PF to degrade intratumoral collagen mesh. Mice were intratumorally injected with 50 µL of PBS, FeCl_3_ (24 µg), propofol (80 µg), Spore (1 × 10^8^ CFU), and Spore@PF (1 × 10^8^ CFU containing 8.5 µg iron ions and 80 µg propofol), respectively. Sirius scarlet staining of tumor tissues collected 48 h after administration showed that Spore@PF induced the most significant reduction of collagen among all the treated groups (Figure 3f). Taken together, these data demonstrated that Spore@PF exhibited a potent collagen degradation capacity both in vitro and in vivo, which were profited from the expression of collagenases as well as the coating‐mediated promotion of bacterial proliferation. As the breakdown of collagen can reduce tumor stiffness and ultimately trigger tumor disintegration, intratumoral delivery of collagenase‐producing *Clostridium* was expected to facilitate tumor therapy.

### 2.4 Metastasis Inhibition Mediated by Spore@PF

**Figure 4:**
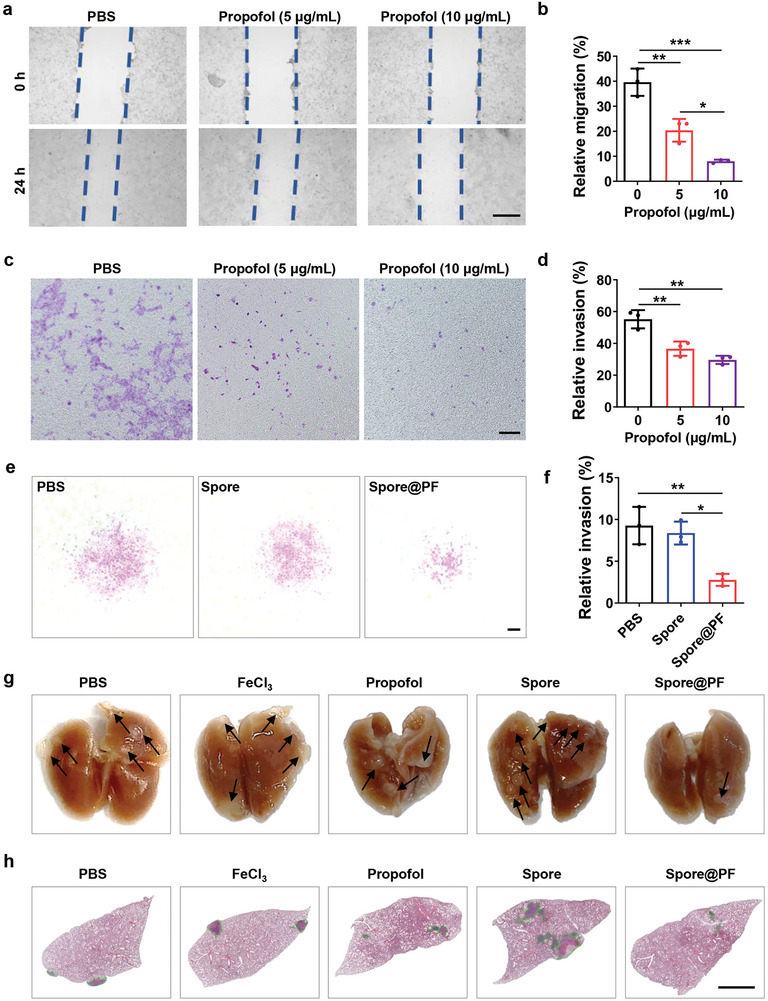
Tumor metastasis inhibited by Spore@PF. a) Microscopic images of scratch‐wounded 4T1 cells after treatment with propofol at various concentrations for 24 h. Scale bar, 500 µm. Blue dotted lines outline the position of the scratch. b) Migration rate of 4T1 cells calculated from scratch‐wounded 4T1 cells after treatment with propofol for 24 h (*n* = 3). c) Microscopic images of 4T1 cells in the basolateral chamber migrated from the 4T1 cell monolayers after co‐cultivation with propofol at various concentrations and d) the calculated relative invasion of 4T1 cells (*n* = 3). Scale bar, 50 µm. e) Microscopic images of 4T1 cells in the basolateral chamber migrated from the 4T1 tumor spheres after co‐cultivation with propofol at various concentrations and f) the calculated relative areas of 4T1 cells (*n* = 3). Scale bar, 50 µm. g) Digital photographs of lungs collected from tumor‐bearing mice 19 days post various treatments. Black arrows indicate metastasis. h) H&E staining of the collected lungs. Green lines delineate tumor tissue. Scale bar, 2 mm. Data are mean ± SD. Statistical analysis was assessed using one‐way ANOVA plus Turkey's post‐test, giving *p* values (*p < 0.05, **p < 0.01, ***p < 0.001).

We first detected the effect of propofol on tumor cell migration using a scratch wound healing assay. A monolayer of 4T1 cells was established and a given amount of propofol was added after scratching by a pipette tip. The images of the cell monolayer were captured at 0 and 24 h after creating the scratch to quantify the migration rate of tumor cells (**Figure** **4**a). As evidenced by Figure 4b, propofol obviously inhibited the cell wound healing, suggesting a significant impediment of tumor cell migration. Transwell assay further proved that fewer tumor cells in the basolateral chamber were migrated from the apical chamber after treatment with 5 or 10 µg mL^−1^ propofol (Figure 4c,d), implying its efficacy to inhibit the invasion of 4T1 cells. In addition to its invasion inhibition effect, we found that the addition of propofol in the cell culture medium could partly inhibit tumor cell growth. As calculated by the cytotoxicity testing using a cell counting kit‐8 (CCK‐8) assay, near 30% 4T1 cells were killed by a concentration of 30 µg mL^−1^ propofol after 24 h incubation (Figure S9, Supporting Information). Having confirmed the ability of propofol to defer tumor cell migration and invasion, 4T1 tumor spheres were employed to evaluate the effect of Spore@PF on inhibiting tumor metastasis. Using a transwell assay, less 4T1 tumor cells were observed in the basolateral chamber in the Spore@PF group compared to the control groups of PBS and Spore (Figure 4e). Crystal violet staining quantified that in contrast to Spore, Spore@PF significantly elevated the inhibition efficiency against 4T1 tumor cell invasion by 3‐times (Figure 4f). To explore the in vivo metastasis inhibition efficacy, orthotopic 4T1 tumor‐bearing mice with an average tumor size of 100 mm^3^ were intratumorally injected with PBS, FeCl_3_, propofol, Spore, and Spore@PF, respectively. Then, lung tissues from the treated mice were harvested and fixed by 4% paraformaldehyde 19 days after treatment. Counting of the small metastases in the lung tissues proved that treatment with Spore indeed promoted lung metastasis, which was furtherly inhibited by Spore@PF (Figure 4g; Figure S10, Supporting Information).

Hematoxylin and eosin (H&E) staining of the lung tissues also showed a markedly decreased level of intrapulmonary metastasis in mice treated with Spore@PF (Figure 4h). Collectively, these findings illustrated that Spore@PF could effectively inhibit tumor metastasis in a propofol‐dependent manner.

### 2.5 Induction of Tumor Cell Ferroptosis

**Figure 5:**
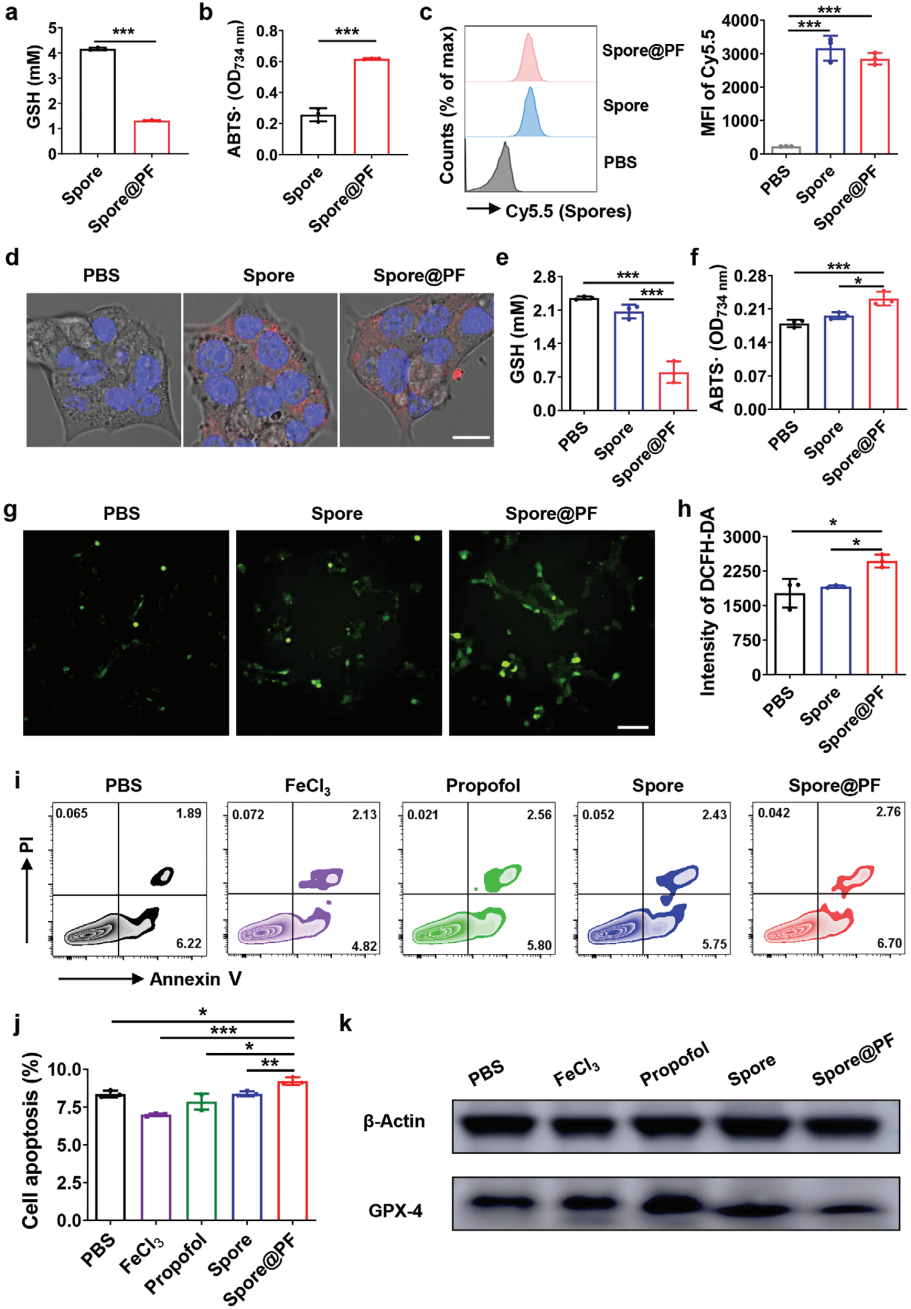
Induction of tumor cell ferroptosis by Spore@PF. a) Level of residual GSH after incubation 2 mm GSH with 1 × 10^8^ CFU of Spore or Spore@PF in BHI medium for 2 h (*n* = 3). b) Antioxidant activity of Spore or Spore@PF measured by a free radical scavenging assay of ABTS (*n* = 3). c) Flow cytometric analysis (*n* = 3) and d) confocal images of 4T1 cells after treatment with Cy5.5‐labeled Spore or Spore@PF (MOI: 10) for 2 h. MFI refers to mean fluorescence intensity. Red and blue channels indicate Cy5.5 and DAPI, respectively. Scale bar, 8 µm. e) Level of GSH in 4T1 cells and f) free radical scavenging assay of 4T1 cells after coculture with Spore or Spore@PF for 2 h (*n* = 3). g) Confocal images and h) fluorescence intensity quantification (*n* = 3) of 4T1 cells after incubation with Spore or Spore@PF (MOI: 10) for 2 h. Cells were labeled with DCFH‐DA probe (green) to detect intracellular ROS. Scale bar, 50 µm. i) Flow cytometric analysis and j) the calculated percentage of Annexin V^+^ cells (*n* = 3) in 4T1 cells after various treatments. Cells were labeled with an Annexin V/PI staining kit. k) Western blot analysis of GPX‐4 expression in 4T1 tumors sampled from mice 48 h after various treatments. β‐Actin was used as an internal control. Data are mean ± SD. Statistical analysis were assessed using two‐tailed Student's *t*‐test for two groups and one‐way ANOVA plus Turkey's post‐test for multiple groups, giving *p* values (*p < 0.05, **p < 0.01, ***p < 0.001).

Having confirmed that Spore@PF were capable of degrading intratumoral collagen mesh and suppressing tumor metastasis, we turned our attention to study whether the carried iron ions could trigger tumor cell ferroptosis via the Fenton reaction. Considering that the reduction of Fe^3+^ ion to its Fe^2+^ form is the initial step to trigger the Fenton reaction,^[^ ^43^ ^]^ we first investigated the response of Spore@PF to GSH, a small‐molecular reducing agent that is abundant in tumor cells.^[^ ^44^ ^]^ Worth noting, the coating of Spore@PF was decomposed after exposure to 2 mm GSH for 10 min (Figure S11, Supporting Information), which was caused by the conversion of Fe^3+^ ions to the Fe^2+^ form (Figure S12, Supporting Information). The reduction of Fe^3+^ ions was further supported by a decrease in GSH concentration after incubation with Spore@PF rather than Spore, as confirmed by both total GSH assay and 2,2′‐azino‐bis(3‐ethylbenzothiazoline‐6‐sulfonic acid) (ABTS) assay (**Figure** **5**a,b). In addition, Fenton reaction caused by Spore@PF was investigated using electron spin resonance (ESR). As displayed in Figure S13 (Supporting Information), strong EPR signals of 5,5‐dimethyl‐1‐pyrroline N‐oxide (DMPO)‐·OH were detected in Spore@PF/GSH/H_2_O_2_ rather than Spore/GSH/H_2_O_2_ system, demonstrating the generation of ·OH induced by Spore@PF. Given the fact that GSH is expressed in the cytoplast,^[^ ^45^ ^]^ it was important to identify whether Spore@PF could enter tumor cells. To verify this, 4T1 cells were co‐cultured with Cy5.5‐labeled Spore or Spore@PF and the tumor cells were collected for flow cytometric analysis after 4 h incubation. As quantified in Figure 5c, a comparable level of Cy5.5 signal was observed between Spore‐ and Spore@PF‐treated cells, indicating that both forms of the spores could be internalized by tumor cells. Confocal images reaffirmed that the spores with or without the coating were capable of entering 4T1 cells (Figure 5d). Correspondingly, Spore@PF effectively depleted GSH and maintained a high level of peroxide in spore‐treated 4T1 tumor cells (Figure 5e,f). Note that the Fenton reaction between Fe^2+^ ions and peroxide generates reactive oxygen species (ROS), which can induce lipid peroxidation and subsequent ferroptosis.^[^ ^46^ ^]^ In line with this notion, 4T1 tumor cells were directly co‐incubated with Spore@PF and subsequently a fluorescent probe of dichlorodihydrofluorescein diacetate (DCFH‐DA) was used to detect the level of intracellular ROS. As shown in Figure 5g,h, co‐incubation with Spore@PF for 2 h substantially induced a higher level of ROS inside 4T1 cells than that of Spore. Glutathione peroxidase 4 (GPX4), a critical inhibitor of lipid peroxidation,^[^ ^47^ ^]^ was obviously reduced in 4T1 cells after incubation with Spore@PF, as assessed by western blot (Figure S14, Supporting Information). The downregulated level of GPX4 implied an amplification of lipid peroxidation, suggesting that Spore@PF could promote ROS production and lipid peroxidation in tumor cells. Then, the effect of Spore@PF on the apoptosis of 4T1 tumor cells was examined by flow cytometric analysis. As clarified in Figure 5i,j, significantly increased proportion of annexin V^+^ cells verified that Spore@PF‐treated tumor cells suffered a higher apoptosis rate than the groups of PBS, FeCl_3_, propofol, and Spore. To demonstrate whether Spore@PF could initiate tumor cell ferroptosis in vivo, orthotopic 4T1 tumor‐bearing mice with an average tumor size of 100 mm^3^ were intratumorally injected with PBS, FeCl_3_, propofol, Spore, or Spore@PF and tumor tissues were collected for western blot analysis 48 h post injection. Expectedly, GPX‐4 was substantially depleted in tumor tissues sampled from Spore@PF‐treated mice rather than the rest groups, verifying the ability to trigger tumor cell ferroptosis in vivo (Figure 5k). Given that Spore@PF could be internalized by tumor cells, we speculated that Spore@PF‐induced depletion of GPX‐4 was largely ascribed to enhanced accumulation of iron ions within the cells. Overall, these findings confirmed the potential of Spore@PF for tumor therapy via activating Fenton reaction‐mediated ferroptosis.

### 2.6 Antitumor Effects of Spore@PF

Encouraged by the selective germination and enhanced proliferation in tumor sites as well as the advantages of degrading intratumoral collagen mesh, inhibiting metastasis, and induction of tumor cell ferroptosis, we further evaluated the therapeutic values of Spore@PF in two different orthotopic 4T1 tumor models. Although the tumor microenvironment is hypoxic, deep regions away from vasculature suffer a more hypoxic milieu,^[^ ^48^ ^]^ which preferably favors the germination of strictly anaerobic spores.^[^ ^49^ ^]^ As a result, we first examined the germination locations of Spore@PF in 4T1 tumors with different volumes to estimate the optimal opportunity for treatment. When the tumor size reaching 100, 300, or 500 mm^3^, 5 × 10^8^ CFU of Spore@PF were injected into the tumors and the numbers of both the spores and germinated spores in the tumor, blood, and major organs were quantified 24 h post injection. Using bacterial plate counting, we found that the gemination rates of the spores in the tumors with the volumes of 100, 300, and 500 mm^3^ were ≈94.76%, 95.26%, and 96.8%, respectively (Figure S15a, Supporting Information), indicating that a more hypoxic milieu in large tumors was beneficial to the germination of the spores. Gram staining revealed that the spores dominantly germinated at the severely hypoxic core regions of the large tumors (Figure S15b–d, Supporting Information). It was worth mentioning that the leakage of germinated spores to the blood and major organs including the liver, spleen, lung, and kidney was limited in comparison to the tumor, highlighting a low risk of pathogenic infection (Figure S16, Supporting Information). Indeed, as claimed in Figure S17 (Supporting Information), treatment with Spore@PF at different doses showed a negligible influence on animal body weight. These data hinted that Spore@PF were safe yet advantageous for tackling intractable large tumors.

**Figure 6:**
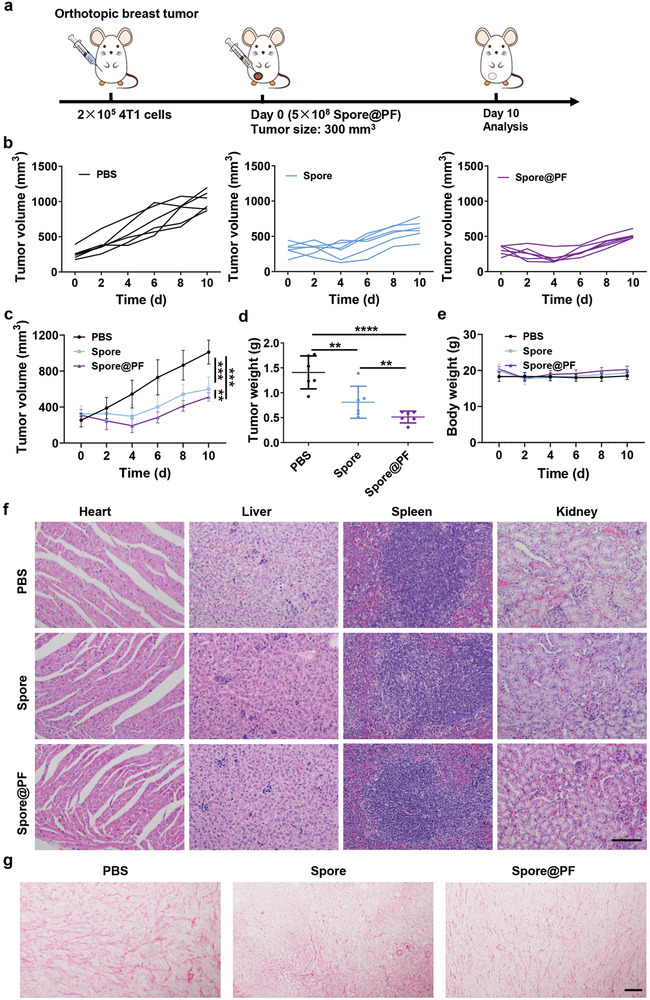
Evaluation of therapeutic efficacy in a regular orthotopic 4T1 model. a) Experimental design. An amount of 5 × 10^8^ CFU of Spore or Spore@PF in 50 µL of PBS was intratumorally injected upon the volume of tumor reaching ≈300 mm^3^. Mice were euthanatized at day 10 for further analysis. b) Individual and c) average tumor growth curves after treatment with PBS, Spore, or Spore@PF (*n* = 6). d) Weights of tumors excised from treated‐mice at day 10 (*n* = 6). e) Variation of body weights of tumor‐bearing mice after different treatments (*n* = 6). f) H&E staining images of the excised major organs including heart, liver, spleen, and kidney. Scale bar, 100 µm. g) Sirius scarlet stained sections of tumors. Scale bar, 100 µm. Data are mean ± SD. Statistical analysis was assessed using one‐way ANOVA plus Turkey's post‐test in multiple groups comparation and Two‐way ANOVA plus Turkey's post‐test was used in tumor growth curves comparation, giving *p* values (**p < 0.01, ***p < 0.001).

Next, mice with orthotopic 4T1 tumor were randomly grouped and intratumorally administered with a signal‐dose of PBS, Spore, or Spore@PF once the size of tumors reaching ≈300 mm^3^ (**Figure** **6**a). The tumor volume and body weight of treated mice were recorded. Mice were euthanatized 10 days after treatment and the tumor and major organs were harvested for related analyses. Despite Spore showed a significant therapeutic effect on tumor inhibition, treatment with Spore@PF could further delay tumor growth (Figure 6b,c; Figure S18, Supporting Information), indicating the synergistic efficacy in impeding tumor progression. Correspondingly, treatment with Spore@PF significantly reduced tumor weight compared to the Spore group (Figure 6d). Then, we analyzed the degree of tissue damage in the tumor using terminal deoxynucleotidyl transferase dUTP nick‐end labeling (TUNEL) and H&E staining. In agreement with the retarded tumor growth, Spore@PF‐treated tumors displayed the most obvious apoptosis and necrosis among all the treated groups (Figure S19, Supporting Information). Of note, Spore@PF treatment showed limited adverse events in mice, reflected by negligible change of body weight and insignificant damage in major organs (Figure 6e,f). As expected, Sirius scarlet staining showed an increased collagen degradation in tumors sampled from Spore@PF‐treated mice, which largely contributed to the therapeutic effects (Figure 6g). The lung metastasis was examined given that tumors with a large size frequently occurred metastasis.^[^ ^50^ ^]^ As confirmed in Figure S20 (Supporting Information), Spore@PF exhibited effective inhibition against lung metastasis compared to the PBS and Spore treatments. Interestingly, the spores or germinated spores were almost absent in the major organs but detectable in the tumor 10 days after injection, validating the specific and durable colonization of Spore@PF in the tumor site (Figure S21, Supporting Information).

**Figure 7:**
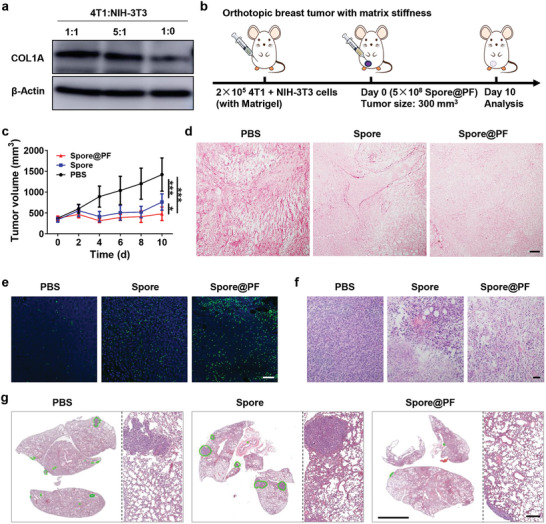
Evaluation of therapeutic efficacy in an orthotopic 4T1 model with stiff matrix. a) Western blot analysis of COL1A expression in tumor spheres consisting of different ratios of 4T1 and NIH 3T3 cells. β‐Actin was used as an internal control. b) Experimental design for in vivo studies. Mice were injected with 50 µL of cell mixture of 4T1 and NIH‐3T3 cells (5:1) along with an equal volume of Matrigel at left mammary fat pad. An amount of 5 × 10^8^ CFU of Spore or Spore@PF in 50 µL of PBS was intratumorally injected upon tumor volume reaching ≈300 mm^3^. Mice were euthanatized at day 10 for further analysis. c) Tumor growth curves of 4T1 tumors (*n* = 6). d) Sirius scarlet staining images of the sectioned tumors. Scale bar, 100 µm. e) TUNEL staining (green) images of the sampled tumors. Cell nuclei were stained with DAPI (blue). Scale bar, 75 µm. f) H&E staining images of the collected tumors. Scale bar, 100 µm. g) H&E staining images of the harvested lungs. Right panels indicate zoomed images. Green lines delineate tumor tissue. Scale bars, 4 mm (left) and 300 µm (right). Data are mean ± SD. Statistical analysis was assessed using two‐way ANOVA plus Turkey's post‐test in multiple groups comparation, giving *p* values (*p < 0.05, ***p < 0.001).

We also assessed the therapeutic effect of Spore@PF in a more malignant tumor model, in which NIH‐3T3 fibroblast cells were co‐inoculated to enhance the density of tumor matrix.^[^ ^51^ ^]^ First, we constructed tumor spheres by mixing 4T1 cells with NIH‐3T3 cells at a range of ratios (1:0, 5:1, and 1:1). Then, the matrix stiffness of these tumor spheres was evaluated by measuring the expression of COL1A (**Figure** **7**a). As predicted, supplement with NIH‐3T3 cells could remarkably increase the COL1A expression and no notable difference was observed between the ratios of 5:1 and 1:1. Thus, we chose the mixture of 4T1 and NIH‐3T3 cells at a ratio of 5:1 to establish the malignant tumor model with stiff matrix (Figure 7b).

Similar to the results obtained by using normal 4T1 tumors, Spore@PF displayed an improved inhibition effect toward malignant 4T1 tumors compared to Spore, as evidenced by the delayed growth and a significantly reduced tumor weight (Figure 7c). Spore@PF also had a superior capacity to degrade collagen in the malignant 4T1 tumor model (Figure 7d). In line with this, treatment with Spore@PF induced higher levels of tumor apoptosis and necrosis compared to the Spore‐treated group (Figure 7e,f). Similarly, malignant tumors with stiff matrix presented obvious metastasis inhibition after Spore@PF treatment (Figure 7g). In addition, mice appeared undetectable body weight fluctuation and tissue damage, indicating the satisfactory tolerance of Spore@PF treatment (Figures S22 and S23, Supporting Information). Briefly, Spore@PF demonstrated the safety and potency for tumor therapy, especially in treating refractory large malignant tumors associated with stiff matrix.

## 3 Conclusion

In summary, we have described the construction of Spore@PF for precision tumor therapy. Spore@PF, dormant collagenase‐producing *Clostridium* individually coated with a Fe^3+^‐propofol network, are prepared by the combination of metal‐phenolic complexation and π‐π stacking interactions. We highlight that Spore@PF not only selectively germinate in tumor sites thanks to the presence of hypoxic immunosuppressive microenvironment, but also rapidly proliferate due to the beneficial effect of the carried Fe^3+^ ions to promote bacterial growth. Meanwhile, the favorable colonization of Spore@PF can produce sufficient collagenases that can efficiently degrade tumor matrix to facilitate tumor therapy. Correspondingly, the loaded propofol is able to inhibit tumor cell migration and invasion to prevent tumor metastasis, while the carried Fe^3+^ ions induce potent Fenton reaction to trigger lipid peroxidation and ultimate ferroptosis of tumor cells as Fe^3+^ ion can be reduced to its Fe^2+^ form by a high level of intracellular GSH. By virtue of the triple antitumor effect, we show that in two different orthotopic models of 4T1 tumor, a single intratumoral injection of Spore@PF significantly retards the progression of established primary tumors with an average size up to ≈300 mm^3^ and also potently suppresses distal lung metastasis. Spore@PF demonstrate the satisfactory safety for tumor treatment, as supported by the high tolerance of tumor‐bearing mice against different doses of the coated spores. Given the versatility of the coating to tune its structure and functionality, this work proposes a unique strategy to develop next‐generation microbial therapeutics that can precisely treat tumors and prevent metastasis.

## 4 Experimental Section

### Materials and Agents

D‐Cycloserine (CAS: 68‐41‐7) and BHI Broth (HB8297), and DCA (HB0284) were purchased from Sangon Biotech (Shanghai) Co., Ltd or Qingdao Hi‐Tech Industrial Park Hope Bio‐Technology Co., Ltd. High glucose Dulbecco's Modified Eagle Medium (DMEM), fetal bovine serum (FBS), and penicillin‐streptomycin antibiotics were purchased from Thermo Fisher Scientific Inc. CCK‐8, TUNEL kit, GSH (S0053), ABTS (S0119) detection kit, and DCFH‐DA kit (S0033S) were purchased from Beyotime. Cy5.5‐NHS ester (S27624) was purchased from Shanghai yuanye Bio‐Technology Co., Ltd. Hydroxyproline assay kit (A030‐3‐1) was purchased from Nanjing Jiancheng Bioengineering Institute Co., Ltd. β‐Actin antibody (clone ARC5115‐01) and polyclonal GPX‐4 antibody were purchased from ABclonal. Polyclonal COL1A antibody was purchased from BOSTER.

### Spore Culture and Purification

Collagenase‐producing *Clostridia* strain was isolated from a soil sample obtained at Shanghai Jiao Tong University School of Medicine Affiliated Renji Hospital (Shanghai, China). *Clostridia* was cultured in a BHI medium supplemented with D‐cycloserine (0.4 g L^−1^) under an anaerobic condition at 37 °C. Viable bacteria were enumerated by counting CFU on DCA plates containing D‐cycloserine (0.4 g L^−1^) under an anaerobic condition at 37 °C.

Bacterial colony was picked from DCA plates and dispersed into 5 mL BHI medium for overnight culture, followed by transferring to 1 L fresh BHI medium for 10 days culture to obtain Spore. The mixture of bacteria and spores was centrifuged at 69 400 × *g* for 50 min and resuspended in 50 mL of sterile PBS. The mixture was then boiled for 20 min at 70 °C and centrifugated at 69 400 × *g* for 50 min to obtain the pellet. After overnight incubation at 4 °C, the pellet was resuspended in 50 mL of sterile ultrapure water and then purified by sucrose density gradient centrifugation. In brief, equal volume sucrose (5 mL) at different densities of 60%, 40%, 20%, and 10% were successionally added to 50 mL Eppendorf tube, and equal mixture was added to the top of tube. The prepared sample was centrifuged at 800 g for 45 min at 4 °C, with a gentle acceleration and deceleration. Spores at the middle layer were collected and washed three times with PBS for further study.

### Cell Culture and Animals

Murine breast cancer cell line 4T1, murine fibroblast cell line NIH‐3T3, and human embryonic kidney cell line 293T were obtained from American Type Culture Collections (ATCC) and cultured in high glucose DMEM medium supplemented with 10% FBS and 100 U mL^−1^ penicillin and 100 µg mL^−1^ streptomycin at 37 °C in a 5% CO_2_ incubator.

Specific‐pathogen‐free female Balb/c mice (6‐8 weeks old) were purchased from Jiesijie (Shanghai, China). All animal procedures were carried out in accordance with the guidelines of Shanghai Medical Experimental Animal Care and approved by the Institutional Animal Care and Use Committee of Shanghai Jiao Tong University (A2020033).

### Preparation and Characterization of Spore@PF

Briefly, 5 × 10^8^ CFU of spores were washed with ultrapure water and resuspended in 1 mL of ultrapure water. Then, 10 µL of propofol (900 µg mL^−1^ in 25% glycerite) with ultrasonic treatment was added into the spore suspension and vortexed for 1 min. Afterward, 10 µL of FeCl_3_ (30 µg mL^−1^) was added and blended into the mixture for 1 min. Spore@PF were centrifugated at 13 000 × *g* for 3 min to obtain a pellet, which was then washed three times with 0.01% Tween‐80 and subsequent three times with ultrapure water.^[^ ^35^ ^]^

The morphology and component analysis of Spore and Spore@PF were observed by FT‐IR (Thermo Scientific Nicolet iN10 MX) spectroscopy, XPS (ESCALAB Xi^+^, Thermo Scientific), AFM (NanoWizard NanoOptics, Bruker) coupled with a DNP‐10 probe (cantilever A, Bruker) under a PeakForce tapping mode, TEM (H7700s, HITACH), and SEM (SU3800, HITACH). Fe mapping was assessed by TEM‐EDS (Talos F200i, Thermo Scientific). The amount of Fe on Spore@PF surface was determined using ICP optical emission spectrometer (Thermo Scientific‐iCAP6300). The propofol loaded on Spore@PF surface was released with acid treatment (1 m hydrochloric acid) for 12 h and measured by UV–vis (Carry‐100, Agilent). The content of propofol was calculated according to a calibration curve. BSA‐FITC (5 µg mL^−1^) was co‐deposited to demonstrate the successful surface coating on Spore by CLSM (TCS SP8, Leica) and FCM (BD FACSVerse, BD Biosciences).

### Survival Rate and Growth Curves of Spore@PF

A number of 1 × 10^7^ CFU of Spores or Spore@PF were resuspended in 1 mL of BHI culture medium supplemented with 0.4 g L^−1^ D‐cycloserine and incubated at 37 °C. The OD value of cultures was detected and recorded at 600 nm every hour by a microplate reader. Meanwhile, the numbers of viable *Clostridia* germinated from Spore and Spore@PF were enumerated on DCA plates after anaerobic incubation overnight at 37 °C, respectively.

### Disassembly of Fe^3+^‐Propofol Network

To assess the degradability of Fe^3+^‐propofol network, Spore@PF were prepared by co‐incubating Spore (5 × 10^8^, 1 mL) with the mixture of FeCl_3_ (30 µg mL^−1^), propofol (900 µg mL^−1^ in 25% glycerite, 10 µL), and doxycycline (100 µg mL^−1^) for 1 min at RT. Then, Spore@PF were resuspended and cultured in solution mimicking the tumor microenvironment (2 mm GSH and 10% FBS) under an anaerobic condition at 37 °C. Spore@PF were centrifugated at 13 000 × *g* for 5 min to obtain the supernatant at the indicated time points. The release profile was assessed by a microplate reader with excitation at 470 nm and emission at 560 nm.

### Collagen Extraction

Extraction and purification of COL1A from rat tails was carried out.^[^ ^52^ ^]^ Briefly, rat tail tendon was teased out from rats and washed with PBS at 4 °C. Then, the collected rat tail tendon was dissolved by 0.02 m acetic acid for 24 h at 4 °C. The sample was centrifuged at 4000 × *g* for 30 min at 4 °C to remove the insoluble components. To achieve the acid‐soluble proteins, the supernatant was purified by dialysis (MWCO, 100 kD) for 24 h at 4 °C. Collagen liquid was dried by freeze‐drying and 10 mg of extracted collagen was dissolved into 1 mL of acetic acid (0.02 m). Extracted or commercial collagen was mixed with protein loading buffer and put in a boiling water bath for 5 min. The samples were analyzed by sodium dodecyl sulfate‐polyacrylamide gel electrophoresis (SDS‐PAGE) gel and Coomassie staining, respectively.

### Collagen Degradation In Vitro

The collagen plate was made up of 0.5% agarose and 10 mg mL^−1^ COL1A dissolved in 0.02 m acetic acid (volume ratio, 1:1). The Spore or Spore@PF were geminated on DCA plates overnight, then one colony was picked up on the collagen plate and cultured for 24 h. The hyalinization of collagen plates was recorded to evaluate collagen degradation.

NIH 3T3 cells were seeded in a 12‐well plate (5 × 10^5^ cells per well) and co‐cultured with 5 × 10^6^ CFU of Spore or Spore@PF for 24 h under an anaerobic condition. Cells were washed with PBS and permeabilized with 0.01% Triton X‐100 for 1 h at 4 °C, followed by staining with Sirius scarlet dye for 1 h at RT. The collagen in cells was dissolved by a mild base solution (0.5% NaOH) and recorded at 540 nm by a microplate reader (BioTek, USA).

The hydroxyproline assay kit was used to detect the products of collagen degradation. Briefly, NIH 3T3 cells were seeded in a 6‐well plate (5 × 10^5^ cells per well) for overnight and then treated with 5 × 10^7^ of Spore or Spore@PF under a hypoxic condition for 24 h. Then, cells were collected, lysed, and detected according to the manufacturer's instructions.

### Cytotoxicity Assay

The cytotoxicity of propofol against 4T1 cells was evaluated by a CCK‐8 assay. 4T1 cells were seeded into a 96‐well plate (0.5 × 10^4^ per well, 100 µL) and cultured overnight. Afterward, various concentrations of propofol were added to the plate for 24 h cultivation. Resultant cells were further incubated with 10% CCK‐8 solution for 3 h at 37 °C and assessed at 450 nm by a microplate reader. The wells containing culture medium and CCK‐8 solution without cells were set as the blank control. The cell viability was determined by comparing the absorption value of propofol‐treated groups to that of the untreated cells.

Briefly, 293T cells were seeded into a 96‐well plate (1 × 10^5^ cells per well, 100 µL) and cultured overnight. Afterward, the prepared Spore@PF with various ratios of spores to cells from 0.1–100 were added to the plate and incubated for 24 h at 37 °C. After washing with PBS for twice, the resultant cells were incubated with 10% CCK‐8 solution for 1 h at 37 °C and assessed at 450 nm by a microplate reader. The cell viability was determined by comparing the absorption value of Spore@PF‐treated groups to that of untreated cells.

### Scratch Wound Healing Assay

Scratch wound healing assay was used to study the cell migration. Briefly, 4T1 cells were seeded into a 6‐well plate (5 × 10^5^ cells per well). When cells reaching a 95% confluence, scratch was produced on the cell monolayer using a pipette tip across the center of the well and the cell culture medium was replaced by fresh FBS‐free medium containing various concentrations of propofol (0, 5, 10 µg mL^−1^) and incubated for another 24 h. Images of cells were captured at 0 and 24 h after creating the scratch and the wound width was measured by Image J. Cell migration was estimated by the following formula of (initial wound width − final wound width)/initial wound width × 100%.

### Invasion Assay

4T1 cells (1 × 10^5^ cells per well) in cell medium supplemented with 1% BSA were seeded in the Matrigel‐coated upper chamber of a 24‐well transwell plate. Cell medium supplemented with 10% FBS was added in the basolateral chamber. After treatment with 5 × 10^6^ of Spore or Spore@PF for 24 h, the cells migrated to the basolateral chamber were fixed by 4% paraformaldehyde for 30 min and stained with 0.1% crystal violet for 20 min at RT. Then, the samples were washed three times with PBS and imaged by a microscope.

4T1 tumor spheres were prepared according to hanging drop technique.^[^ ^53^ ^]^ Briefly, 20 µL of 4T1 cells (2 × 10^5^) mixed with 0.24% carboxymethyl cellulose was pipetted onto the lids of 100 mm dishes and inverted over dishes containing 10 mL of PBS. Hanging drips were incubated for ≈5 to 7 days to form tumor spheres by adequate gravity‐sedimentation. Tumor spheres were collected and co‐cultured in the upper chamber with Spore or Spore@PF for 24 h under an anaerobic condition. The cells migrated to the basolateral chamber were fixed, stained, and imaged as above description.

### Tumor Infiltration and Cellular Uptake of Spore@PF

To assess cellular uptake of Spore@PF, 4T1 cells (5 × 10^5^) were seeded in 3.5 mm glass bottom dish overnight and incubated with Cy5.5‐labeled Spore (5 × 10^6^) or Spore@PF (5 × 10^6^) in 1 mL of basic cell culture medium. After cultivation for 2 h, cells were washed three times with PBS and observed by CLSM. For FCM analysis, treated 4T1 cells were dissociated by 0.25% trypsin for 1 min at 37 °C. To confirm the infiltration of Spore@PF, 4T1 tumor spheres were collected and stained with Hoechst 33 342 (10 µg mL^−1^) for 20 min at 37 °C. Then, 4T1 tumor spheres were incubated with fresh basic cell culture medium supplemented with Cy5.5‐labeled Spore or Spore@PF (1 × 10^7^) for 4 or 24 h. The fluorescence intensity of spheroids, Spore or viable *Clostridia* was observed by CLSM.

### Colorimetric Phenanthroline‐Based Assay for the Quantitation of Fe^2+^

1,10‐Phenanthroline (0.5%) dissolved in hydrochloric acid solution (0.1 mm) was employed to quantify the conversion of Fe^3+^ ions to the Fe^2+^ form of Spore@PF after exposure to GSH. Fresh FeSO_4_ (0, 40, 200, 400 µm) was used for standard calibration curve of Fe^2+^. Spore or Spore@PF (1 × 10^10^) were resuspended with 2 mm GSH for 10 min at RT. The supernatant was collected after centrifugation at 10 000 × *g* for 3 min and then incubated with detection solution (100 µL) supplemented with FeSO_4_ and fresh Ferrozine solution (10 µL) at 37 °C for 10 min. The resultant solution was assessed at 570 nm by a microplate reader.

### Total GSH Detection

The concentration of GSH was assessed by total GSH assay. Briefly, Spore or Spore@PF (1 × 10^8^) were incubated with 2 mm GSH for 10 min and the supernatant was collected. To detect the GSH level in cells, 4T1 cells (5 × 10^5^) were seeded into a 6‐well plate for overnight cultivation. Spore or Spore@PF (5 × 10^6^) were added to the well and the mixture was incubated at 37 °C for 2 h. After washing three times with cold PBS, 4T1 cells were collected and lysed in 200 µL of PBS. The prepared sample was centrifuged at 12 000 × *g* for 10 min at 4 °C and the supernatant was separated. Subsequently, GSH detection working solution (150 µL) containing GSH reductase and DNTB solution was added to 50 µL of the resultant supernatant, and the mixture was incubated for 25 min at RT. After adding 50 µL of 0.5 mg mL^−1^ NADPH solution, the absorbance of the sample was measured at 412 nm by a microplate reader.

### ABTS Radical Scavenging Assay

The ABTS radical scavenging assay is a colorimetric assay based on the formation of radical cation ABTS•^+^. Briefly, the radical cation ABTS•^+^ solution was prepared by mixing ABTS and K_2_S_2_O_8_, and the resultant solution was kept in the dark for 16 h at RT. Spore or Spore@PF (1 × 10^8^) were incubated with 2 mm GSH for 10 min and the supernatant was collected. To detect the radical scavenging in cells, 4T1 cells (5 × 10^5^) were seeded into a 6‐well plate for overnight cultivation and subsequently co‐cultured with Spore or Spore@PF (5 × 10^6^) at 37 °C for 2 h. The resultant 4T1 cells were washed and lysed in 200 µL of cold PBS. The prepared sample was centrifuged at 12 000 × *g* for 5 min at 4 °C and the supernatant was collected. Then, ABTS•^+^ solution (200 µL) was added to 10 µL of the resultant supernatant, and the mixture was incubated in the dark for 5 min at RT. The absorbance of the sample was measured at 734 nm by a microplate reader.

### Fenton Reaction Detection

ESR was conducted to determine the generation of·OH in Spore@PF/GSH/H_2_O_2_ Fenton reaction system. Spore or Spore@PF (5 × 10^8^ CFU, 100 µL) were incubated with GSH (2 mm) for 10 min at RT and then mixed with H_2_O_2_ (100 µm) for detecting the ESR signal of·OH with the assistant of DMPO as a trapping reagent.

### ROS Assay

4T1 cells (5 × 10^5^) were seeded in 3.5 mm glass bottom dish overnight and incubated with Spore or Spore@PF (5 × 10^6^) in basic cell culture medium at 37 °C for 2 h. After washing three times with PBS, the cells were incubated with 5 µm DCFH‐DA work solution for 30 min at 37 °C. After washing three times with PBS, the fluorescence of DCFH‐DA in cells was observed by CLSM. For quantification of ROS, the fluorescence of DCFH‐DA was detected by a microplate reader with excitation at 485 nm and emission at 535 nm.

### Apoptosis Assay

4T1 cells were seeded in a 12‐well plate (1.5 × 10^5^ cells per well) and cultured overnight at 37 °C. Cells were then treated with PBS, equivalent FeCl_3_ (0.48 µg mL^−1^), equivalent propofol (1.6 µg mL^−1^), Spore (2 × 10^6^ CFU), and Spore@PF (2 × 10^6^ CFU), respectively, under an anaerobic condition for 24 h. Cells were harvested and detected according to the manufacturer's instructions by FCM analysis.

### Germination Rate of Spore@PF In Vivo

The germination rate of Spore@PF in tumors was evaluated by mice with orthotopic breast cancer. Briefly, mice were injected with 50 µL of 4T1 cells (2 × 10^5^) into the left mammary fat pad. Tumors were allowed to grow until they reached a size of 100, 300, or 500 mm^3^. Subsequently, the mice were intratumorally administered with 5 × 10^8^ CFU of Spore or Spore@PF. A portion of tumor tissues and other major organs were collected and homogenized, which were spread on DCA plates for overnight incubation at 37 °C before counting. The CFU counts of Spore were enumerated on DCA plates after treatment with 75% alcohol for 30 min. Another part of tumor tissue was fixed and sliced for Gram staining to trace the germinated spores.

### Collagen Degradation Mediated by Spore@PF In Vivo

When tumors reaching 100 mm^3^, mice were randomly divided to five groups and treated with 50 µL of PBS, FeCl_3_ (24 µg), propofol (80 µg), Spore (1 × 10^8^ CFU), or Spore@PF (1 × 10^8^ CFU), respectively. Tumors were collected after treatment at 48 h, followed by fixation and section for Sirius scarlet staining.

### Therapeutic Efficacy In Vivo

Mice were injected with 50 µL of cell mixture of 4T1 and NIH‐3T3 cells (5:1) along with equal volume of Matrigel at left mammary fat pad to mimic the malignant tumor model with matrix stiffness. When tumors reaching 300 mm^3^, tumor‐bearing mice were intratumorally injected with 50 µL of PBS, Spore (1 × 10^8^ CFU), and Spore@PF (1 × 10^8^ CFU), respectively. Tumor growth and body weight were monitored at a 2‐day interval. The tumor volume was estimated according to the formula of 0.5 × length × (width)^2^. Tumor tissues and lungs were excised at day 10 post‐administration. After measuring tumor weight, tumor tissues were fixed and sliced for TUNEL, H&E, or Sirius scarlet staining. Lungs were fixed in 4% PFA and lung metastasis nodules were then manually enumerated. To further assess intrapulmonary metastasis nodules, lungs were embedded and sliced for H&E staining.

### Western Blot

All cell or tissue lysates were separated by 4–12% SDS‐PAGE. The proteins in gels were transferred to polyvinylidene difluoride membranes. Membranes were washed with tris buffered saline with 0.1% Tween 20 (TBST). Blocking was carried out by Fast Blocking Western for 10 min at RT. Then, the membranes were incubated with anti‐GPX4 (1:1000), anti‐COL1A (1:200), or anti‐Actin (1:5000) overnight at 4 °C, followed by washing and incubating with secondary antibody. Images of protein bands were visualized by an electrogenerated chemiluminescence system (GE&Amersham Imager 680R).

### Statistical Analysis

GraphPad Prism 8.0 was employed for figure generation and statistical analysis. All data were presented as mean ± standard deviation (SD). Statistical differences for comparison of two groups were evaluated by unpaired two‐tailed Student's *t*‐test. Statistical differences for comparison of multiple groups were performed using one‐way ANOVA plus Turkey's post‐test. Statistical differences of multiple curves were analyzed by two‐way ANOVA plus Turkey's post‐test for multiple comparisons. Significance was defined as a *p* value < 0.05 in all experiments (*p < 0.05, **p < 0.01, ***p < 0.001, ****p < 0.0001). NS represents no significance.

## Conflict of Interest

The authors declare no conflict of interest.

## Supporting information



Supporting Information

## Data Availability

The data that support the findings of this study are available from the corresponding author upon reasonable request.
