# Supplementary material for: Coating Dormant Collagenase‐Producing Bacteria with Metal‐Anesthetic Networks for Precision Tumor Therapy
Source: Adv Sci (Weinh). 2024 Sep 18;11(42):2407402. doi: 10.1002/advs.202407402 (PMC11558152; doi:10.1002/advs.202407402)
Supplement: Supplementary file 1 — Supporting Information [file ADVS-11-2407402-s001.docx]

Supporting Information

**Coating Dormant Collagenase-Producing Bacteria with Metal-Anesthetic Networks for Precision Tumor Therapy**

*Qiuju Han^1,2#^, Fengmin Yang^1#^, Mian Chen^1,2^, Mengmeng Zhang^1^, Lu Wang^1*^, Hongxia Wang^3*^, Jinyao Liu^1,2*^, Zhenping Cao^1*^*

^1^Shanghai Key Laboratory for Nucleic Acid Chemistry and Nanomedicine, Institute of Molecular Medicine, State Key Laboratory of Systems Medicine for Cancer, Shanghai Cancer Institute, Renji Hospital, School of Medicine, Shanghai Jiao Tong University, Shanghai 200127, China.

^2^School of Chemistry and Chemical Engineering, Shanghai Jiao Tong University, Shanghai 200240, China.

^3^Department of Medical Oncology, Fudan University Shanghai Cancer Center, Department of Oncology, Shanghai Medical College, Fudan University, Shanghai 200032, China.

^#^These authors contributed equally to this work. ^*^Correspondence: (Z.C.) caozhenping@renji.com; (J.L.) jyliu@sjtu.edu.cn; (H.W.) whx365@126.com; (L.W.) biowl@sjtu.edu.cn

**Supplementary Figure 1-20**


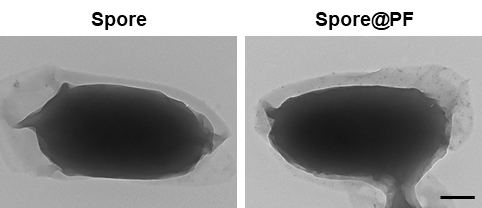


**Fig. S1** Representative TEM images of Spore and Spore@PF. Scale bar, 200 nm.


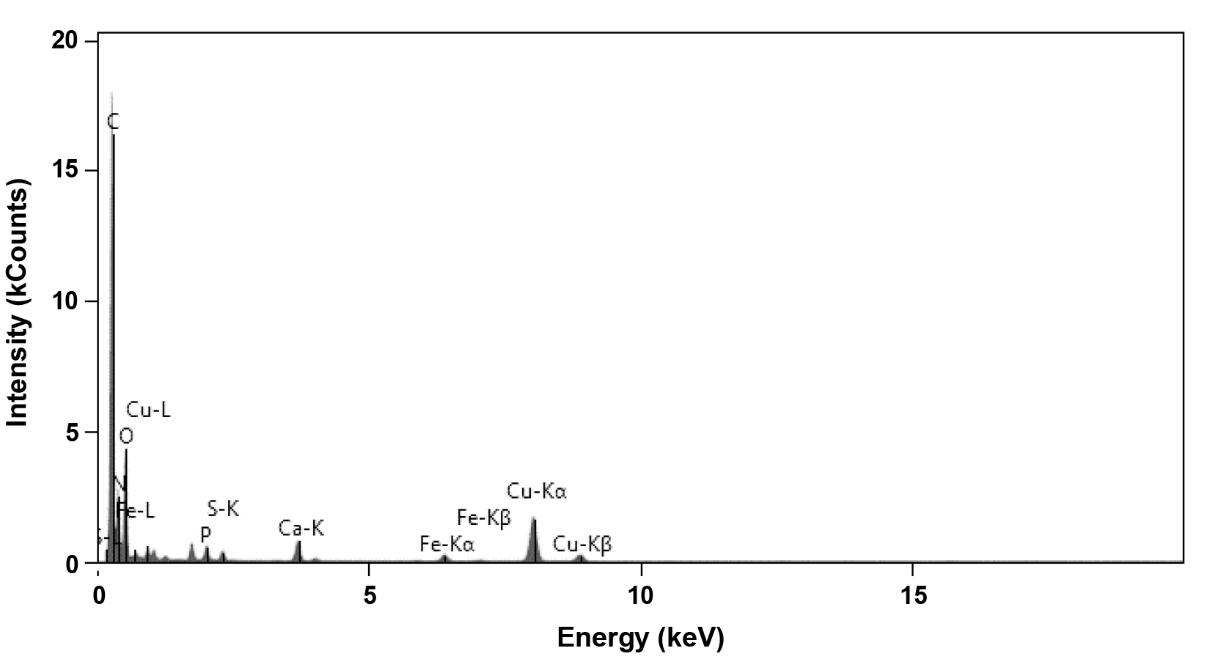


**Fig. S2** TEM-EDS elements energy spectrum of Spore@PF.


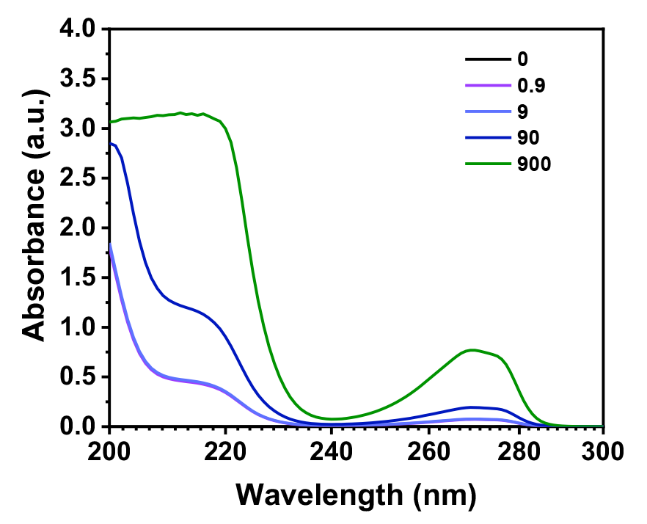


**Fig. S3** Quantitation of propofol loaded on Spore@PF. UV-Vis spectra of propofol with different concentrations.


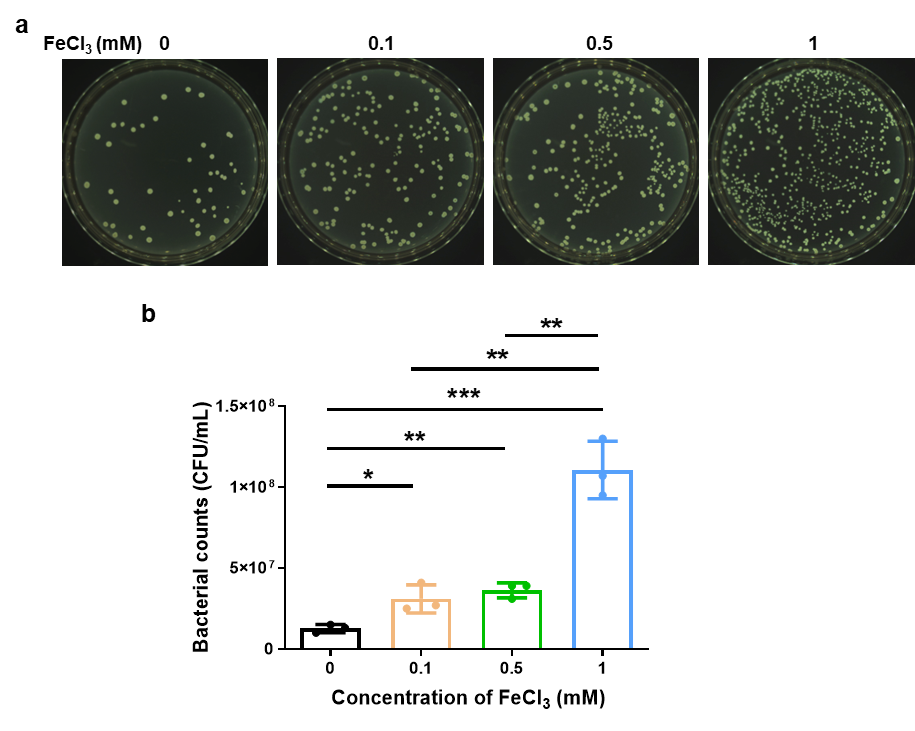


**Fig. S4** Proliferation after the germination of Spore co-cultured with FeCl_3_ at various concentration for 12 h. (**a**) Representative photographs of DCA plates in equal dilution. (**b**) Quantification of DCA plates (*n* = 3). Data are mean ± SD. Statistical analysis was assessed using one-way ANOVA plus Turkey’s post-test. **P* < 0.05, ***P* < 0.01, ****P* < 0.001.

**Fig. S5** Disassembly of Fe^3+^-propofol network on spore surface by determining the release of loaded doxycycline at 37 °C (*n* = 3). Data are mean ± SD.

**Fig. S6** Cell viability of 293T cells measured by CCK-8 assay after incubating with different concentrations of Spore@PF (MOI: 0, 0.1, 0.5, 1, 5, 10, 50, or 100) for 24 h (*n* = 6). Data are mean ± SD.

**Fig. S7** Number of germinated spores in the tumor site and main organs (heart, liver, spleen, lung, kidney) 24 h post intratumoral injection with Spore@PF. A number of 1 × 10^8^ CFU of spores were injected intratumorally once the size of the tumor reaching 100 mm^3^ (*n* = 3). Data are mean ± SD.


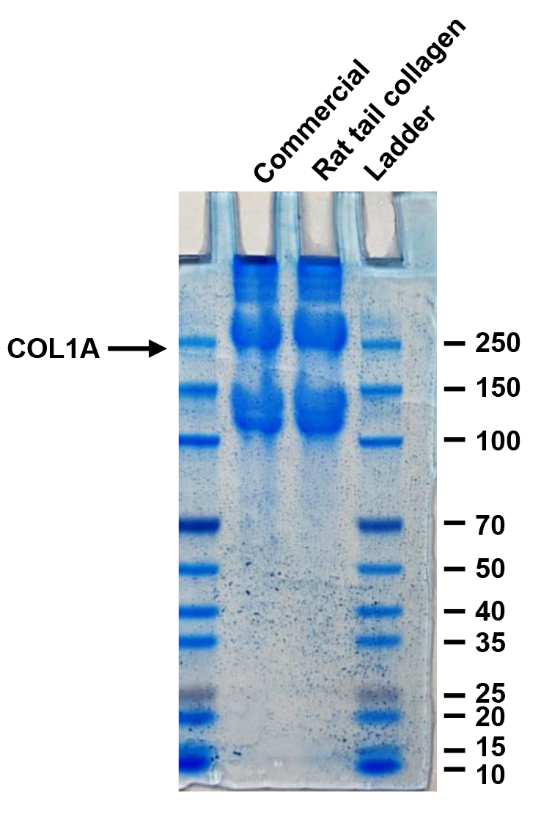


**Fig. S8** Commas staining of SDS-PAGE of collagen. Numbers indicate estimated molecular weight in kilodaltons (kDa).


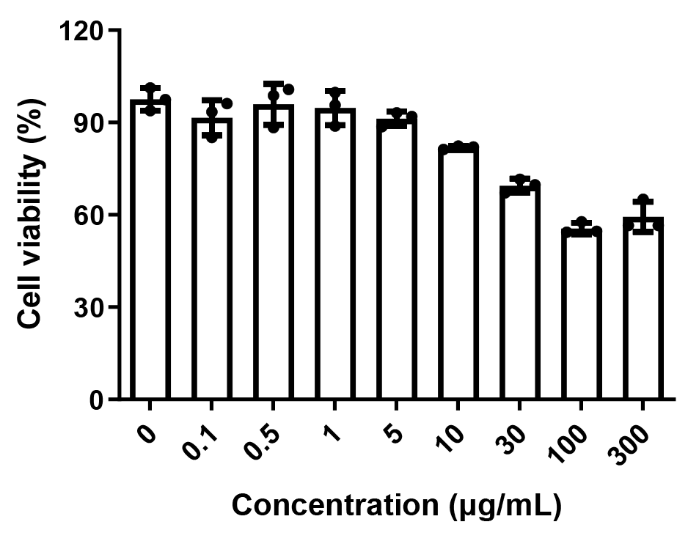


**Fig.** **S9** Cell viability of 4T1 cells after treatment with various concentrations of propofol for 12 h (*n* = 3). Data are mean ± SD.


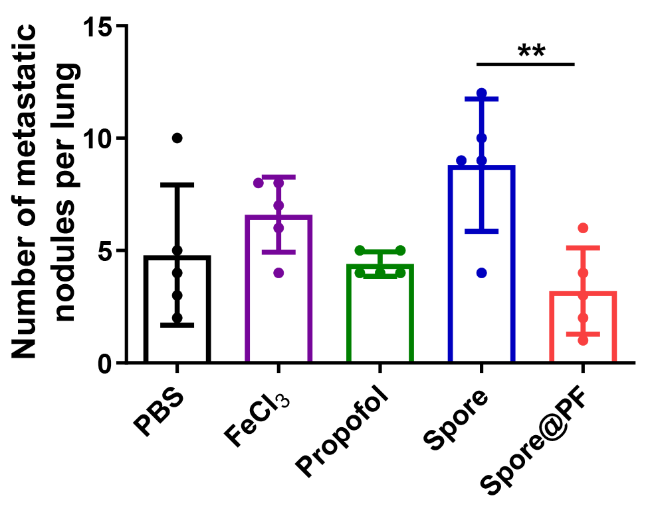


**Fig. S10** Quantification of metastasis in the lungs of tumor-bearing mice treated with PBS, FeCl_3_, propofol, Spore, or Spore@PF at day 19 (*n* = 5). Data are mean ± SD. Statistical analysis was assessed using one-way ANOVA plus Turkey’s post-test. ***P* < 0.01.


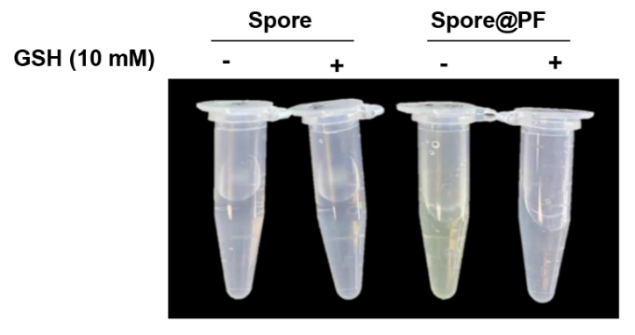


**Fig. S11** Representative digital photographs of the disintegrated coating on Spore@PF after treatment with GSH solution for 10 min at RT.


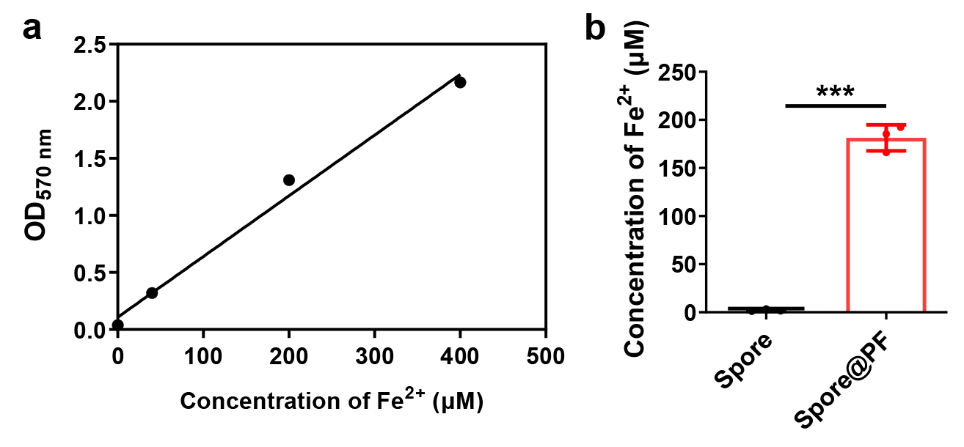


**Fig. S12** Conversion of Fe^3+^ ions to the Fe^2+^ form induced by GSH. (a) Calibration curve of propofol concentration in corresponding to the value of optical density at 570 nm. (b) Concentrations of Fe^2+^ of Spore and Spore@PF after exposure to 2 mM GSH for 24 h (*n* = 3). Data are mean ± SD. Statistical analysis was assessed using two-tailed Student’s *t*-test. ****P* < 0.001.


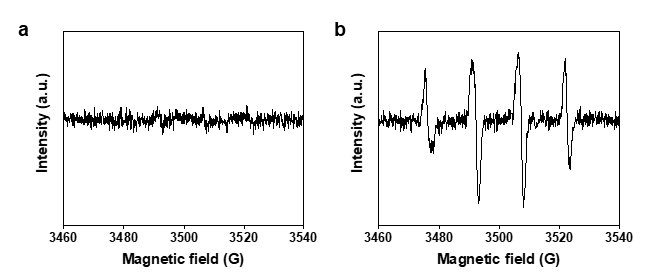


**Fig. S13** ESR spectra of ·OH in the (a) Spore/GSH/H_2_O_2_ and (b) Spore@PF/GSH/H_2_O_2_ Fenton reaction system captured by spin-trapping agent of DMPO.


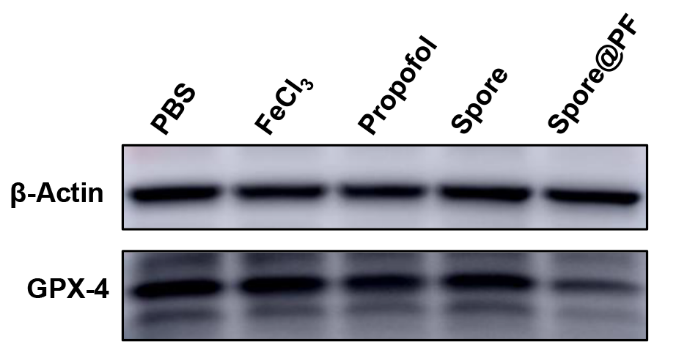


**Fig.** **S14** Western blot analysis of GPX-4 expression in 4T1 cells treated with PBS, FeCl_3_, propofol, Spore or Spore@PF for 12 h. β-Actin was used as an internal control.


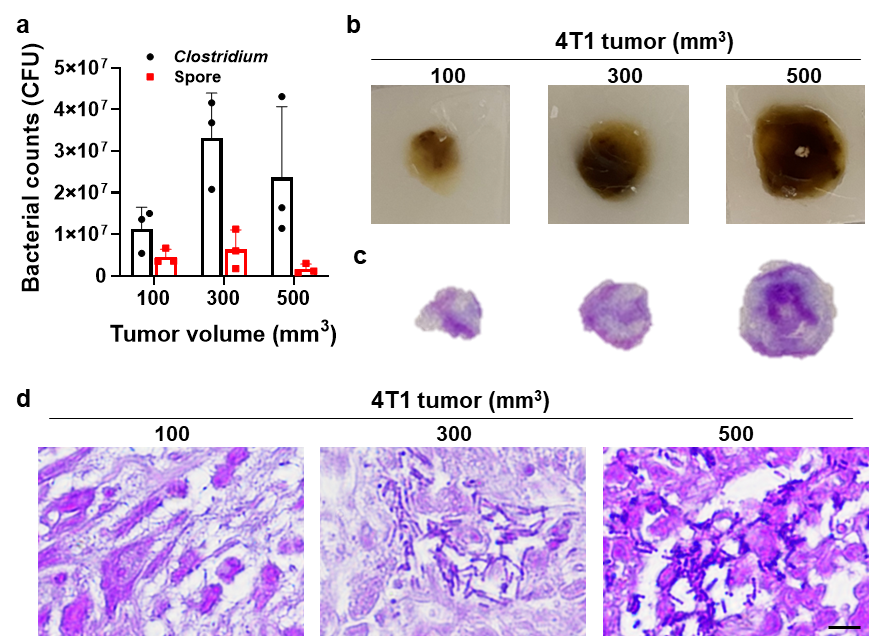


**Fig.** **S15** Spore retention in 4T1 tumors. (**a**) Quantification of Spore@PF in tumors at 24 h post-injection by bacterial plate counting (*n* = 3). Data are mean ± SD. (**b**) Representative digital photographs of the collected tumors. (**c**) Representative images of Gram staining of tumor tissues. (**d**) Enlarged images from (c). Scale bar, 10 μm.


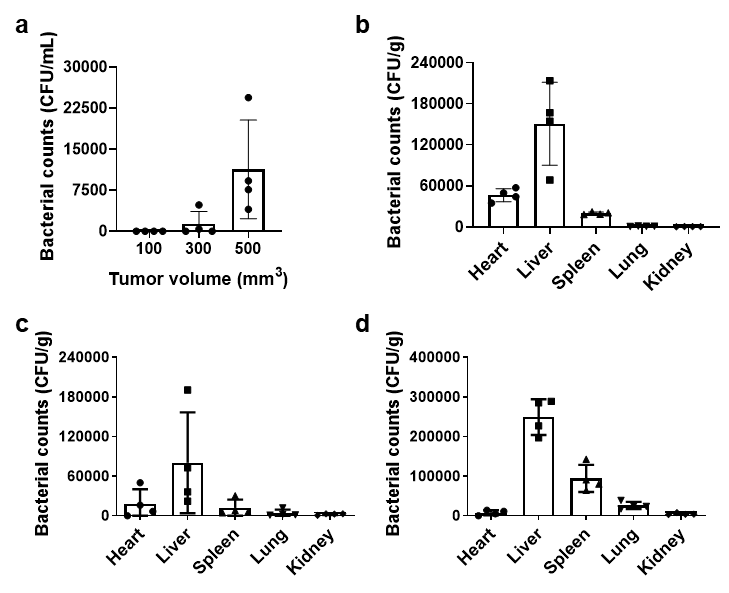


**Fig. S16** Spore or germinated spores in mice (*n* = 4). (a) Spore or germinated spores in the blood of mice with various tumor volumes (100, 300, or 500 mm^3^). Spore or germinated spores in the heart, liver, spleen, lung, and kidney of tumor-bearing mice with a volume of (b) 100, (c) 300 or (d) 500 mm^3^. Data are mean ± SD.


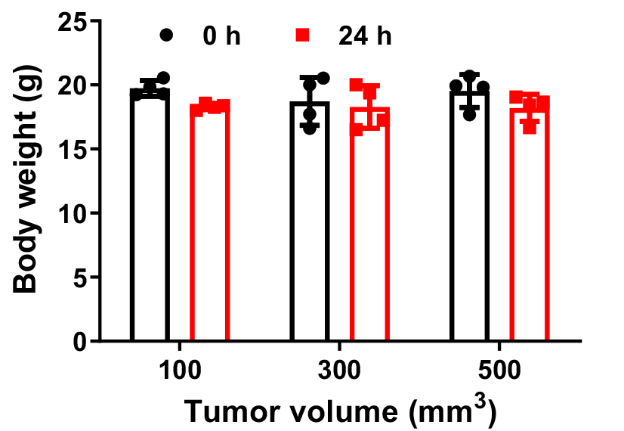


**Fig. S17** Weight of tumor-bearing mice with various tumor volumes at 0 and 24 h after treatment with Spore@PF (*n* = 4). Data were mean ± SD.


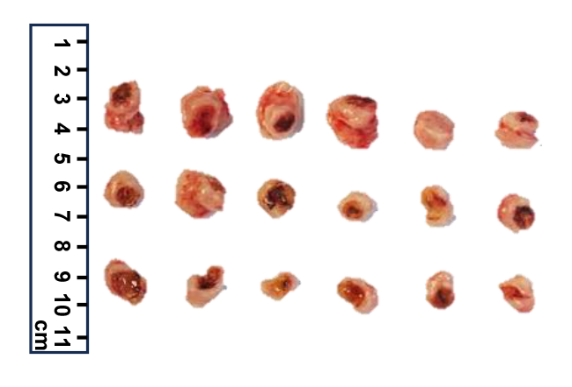


**Fig.** **S18** Digital photographs of tumors excised from treated-mice at day 10 (*n* = 6).


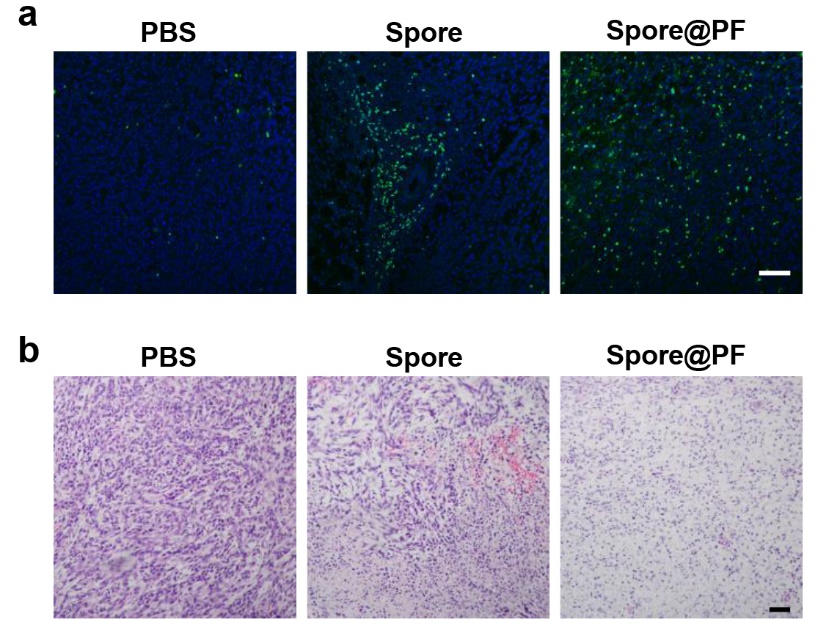


**Fig. S19** Representative confocal images of (a) TUNEL (green) and H&E staining of tumors excised from mice treated with PBS, Spore, or Spore@PF at day 10. Cell nuclei were stained with DAPI (blue). Scale bars, 75 μm (a) and 200 μm (b).


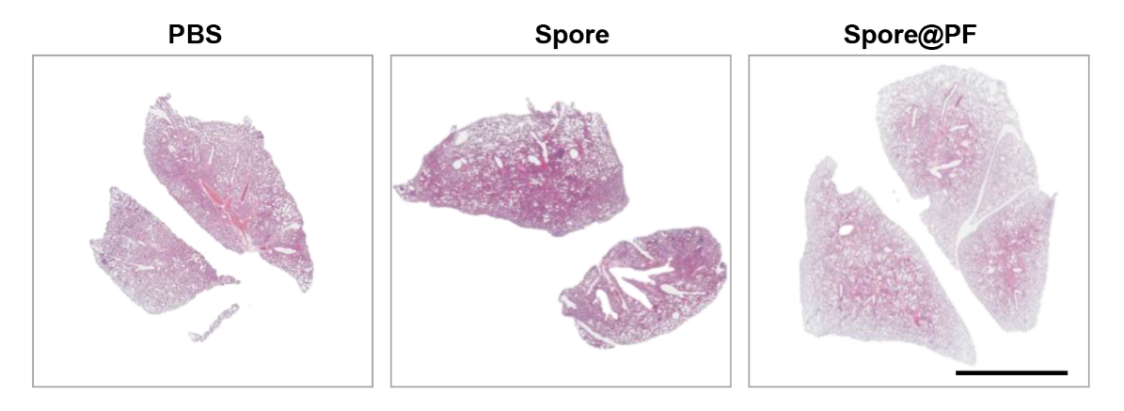


**Fig. S20** Representative images of H&E staining of lungs excised from mice treated with PBS, Spore, or Spore@PF at day 10. Scale bar, 4 mm.

**Fig. S21** Quantification of Spore or germinated spores in the heart, liver, spleen, lung, and kidney excised from mice treated with Spore or Spore@PF at day 10 (*n* = 5). Data are mean ± SD.

**Fig. S22** Body weight variation of mice bearing stiffness 4T1 tumors after treatment with PBS, Spore, or Spore@PF (*n* = 6). Data are mean ± SD.


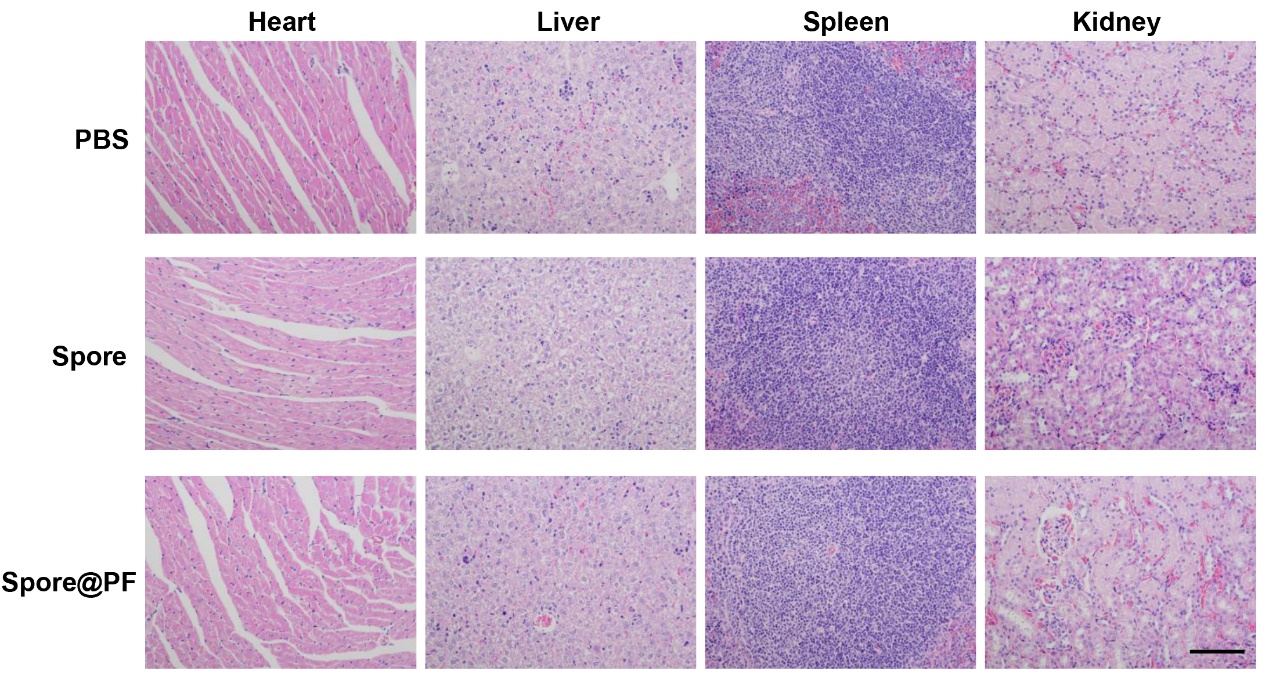


**Fig. S23** Representative images of H&E staining of the major organs including heart, liver, spleen, and kidney from mice bearing stiffness 4T1 tumors after treatment with PBS, Spore, or Spore@PF. Scale bar, 100 μm.
